# Effects of Body Mass Index on Brain Structures in the Elderly: Longitudinal Analyses

**DOI:** 10.3389/fendo.2022.824661

**Published:** 2022-06-03

**Authors:** Hikaru Takeuchi, Ryuta Kawashima

**Affiliations:** ^1^Division of Developmental Cognitive Neuroscience, Institute of Development, Aging and Cancer, Tohoku University, Sendai, Japan; ^2^Smart Aging Research Center, Tohoku University, Sendai, Japan; ^3^Department of Advanced Brain Science, Institute of Development, Aging and Cancer, Tohoku University, Sendai, Japan

**Keywords:** BMI, brain structures, dementia, cognitive functions, longitudinal

## Abstract

The relationship between obesity and neurocognitive consequences is complex. Here we investigated associations between body mass index (BMI) and subsequent changes in brain structures, cognitive changes, and the onset of dementia after adjustment of a wide range of potential confounding variables using a large prospective cohort data of UK Biobank. After correcting for confounding factors, higher BMI was associated with greater retention in visuospatial memory performance (decline in error numbers) [beta = -0.019 (CI:-0.027~-0.016), N = 39191], increase in depression tendency scores [beta = 0.036(0.027~0.045)] as well as decreased risk of incident dementia [increasing BMI by 1 is associated with HR of 0.981 (CI:0.969~0.992), N = 398782], but not changes in fluid intelligence or reaction time. Whole brain multiple regression analyses (volumetric analyses: N = 1253, other analyses: N = 1241) revealed positive associations between BMI and subsequent changes in regional gray matter volume (rGMV) in multiple areas, regional white matter volume changes in widespread white matter (WM) tracts, fractional anisotropy changes in several tracts, and intracellular volume fraction (ICVF) and orientation dispersion (OD) in widespread areas, and isotropic volume fraction (ISOVF) in a few areas, and negative associations between BMI and subsequent changes in rGMV in the bilateral medial temporal lobe areas, mean, axial and radial diffusivity, and ISOVF in widespread areas. These results are mostly consistent with the view that less BMI precedes greater neurocognitive aging or atrophy, with a few exceptions including OD findings and the rGMV finding of the medial temporal lobes as most of significant longitudinal associations of higher BMI were opposite to those seen in higher age and dementia. Future epidemiological studies should consider separating effects of higher BMI itself from potential confounders.

## Introduction

Obesity is one of the major health problems in modern society; 2.1 billion people are obese or overweight (1). Obesity promotes co-morbid diseases (2) and reduces life expectancy by a few years (3). Specifically, obesity is strongly associated with an increased risk of cardiovascular diseases and stroke (2).

On the other hand, the relationship between obesity and risk of developing dementia and long-term cognitive changes are more complex. Meta-analyses of longitudinal studies have reported contradicting findings regarding the risk of dementia in obese subjects. For instance, one meta-analysis concluded that obesity in middle age (35-65 years) is associated with increased risk of dementia later in late life (4), But more recent studies with huge sample size showed, that obesity in both middle age and old age was associated with a lower risk of later onset of dementia (5–7). As for cognitive functions, studies either found the associations of greater BMI in the middle and late life and subsequent decline in cognitive decline or failed to show them (8). But one study showed that accounting for effects of education level and occupation and background substantially weaken such associations (9). In addition, obesity has a relationship with affective state, and Luppino et al. (10)’s meta-analysis showed that obesity is associated with an increased risk of subsequent depression and that there is a reciprocal relationship between obesity and depression.

Numerous neuroimaging studies of BMI and obesity have been performed. Meta-analyses of cross-sectional studies support that obesity or high BMI are associated with less gray matter volume in the medial prefrontal cortex and left temporal pole (11), a decrease in the hippocampal region of interest (ROI) volume (12), and decline in gray matter (GM) ROI volumes and increase in white matter (WM) ROIs’ volumes (13). Cross-sectional studies of microstructural properties measured by diffusion tensor imaging (DTI) revealed that BMI is associated with lower fractional anisotropy (14) and lower mean diffusivity in the GM area of the dopaminergic system (15).

However, these studies have not clarified the relationship between BMI and longitudinal changes in the microstructural properties of brain. In addition, the relationship between BMI and subsequent changes in regional GM volume (rGMV) and regional WM volume (rWMV) have not been clarified using voxel-by-voxel analyses that adjusted for brain structures at the baseline. The purpose of this study is to elucidate these issues. Additionally, in this study, we used the UK Biobank data, which is rich in both sample size and type, to evaluate whether higher BMI is associated with lower subsequent cognitive decline and lower risk of dementia simultaneously, after adjusting for sufficient potential confounding variables.

We set two opposing hypotheses: our first hypothesis was higher BMI would be associated with subsequent neuroprotective changes. Specifically, we hypothesized lower BMI would be associated with the subsequent changes in brain structures that are seen in aging and dementia (e.g., decline rGMV, rWMV, FA, ICVF increase of MD/AD/RD/ISOVF) (16, 17). The second hypothesis is the opposite. These two hypotheses are based on conflicting evidence of previous neurocognitive studies of obesity and BMI as introduced above.

## Methods

### Participants

**Figure 1:**
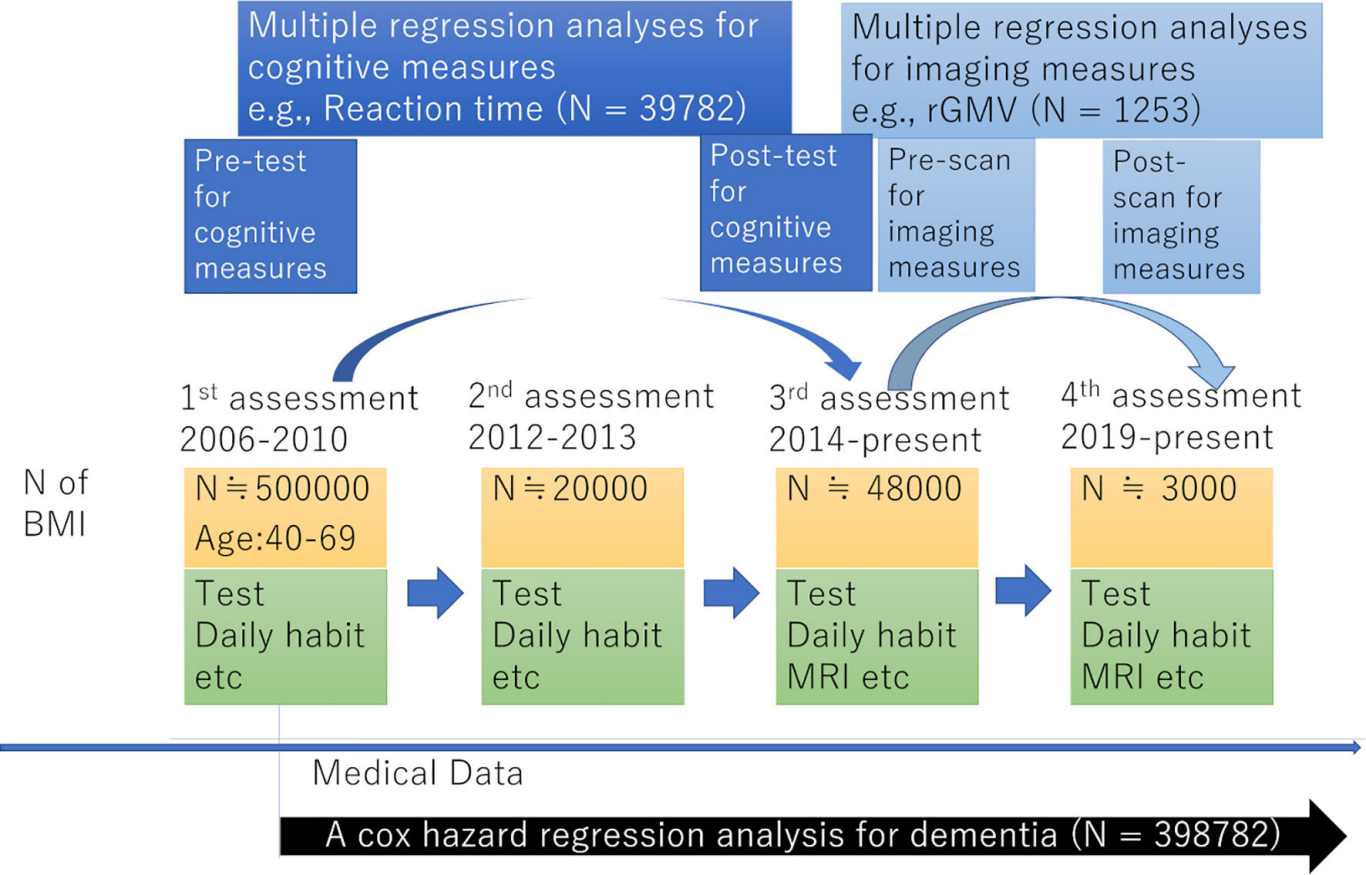
A schema of the analyses of this study and its association with UK Biobank.

The present study used data from the UK Biobank, which was obtained from a prospective cohort study of a middle-aged population in the United Kingdom and the procedures of which have been described elsewhere (http://www.ukBiobank.ac.uk/wp-content/uploads/2011/11/UK-Biobank-Protocol.pdf). Approval for these experiments was obtained from the North-West Multi-center Research Ethics Committee (reference number: 11/NW/0382) and written informed consent was obtained from each participant. Briefly, the participants went to one of twenty-two assessment centers throughout the UK for data collection, with baseline data obtained from 502,505 participants. Our study included data for this cohort obtained at the first assessment visit (2006-2010), the first imaging data collection visit, which corresponded to the third assessment visit (2014-present), and the follow-up visit for imaging data collection, corresponding to the fourth assessment visit (2019-present). The study schema is presented in **Figure 1**.

This research was conducted using the UK Biobank resource under application number 56726. Following analyses were conducted from the data of subjects whose all dependent and independent data was available.

### BMI Measurement

Body weight was measured using Tanita BC418MA scales. Height was measured using a Seca height measure. BMI was calculated from measured height and weight.

### Sociodemographic and Lifestyle Measurements Used as Covariates

Self-reported gender data was used. From the database, the neighborhood-level socioeconomic status at recruitment (cov1), education level at recruitment (cov2), household income (cov3), current employment status (cov4), metabolic equivalent of task hours (MET) (cov5), number in household (cov6), current tobacco smoking level (cov7), current alcohol drinking level (cov8), use of statin (cov9), the diagnosis of diabetes (cov10), ethnicity (white or not)(cov11), length of TV viewing (cov12) are included as covariates. For additional details, refer to the **Supplementary Methods** section.

### Cognitive Measures

Cognitive measures were administered at all visits. Briefly, tests were administered through a computerized touch-screen interface at each assessment center. In this study, we used data of visuospatial memory performance, fluid intelligence and reaction time.

Visual short-term memory consists of the basic mechanisms for retention and manipulation of visuospatial information not already present in the environment, and is an important function for orientation and navigation in the environment, and organization and retention of visuospatial representations of the visuospatial world (18, 19). Fluid intelligence refers to the basic processes involved in reasoning and other cognitive activities that require minimal prior learning, culture, or education. Fluid intelligence involves various cognitive activities and is typically measured by nonverbal reasoning, despite the importance of ability to solve abstract reasoning problems (20).

Visuospatial memory was measured by the “pairs-matching’ task. In this test, participants were asked to memorize the positions of six card pairs, and then match them from memory while making as few errors as possible. Scores on the pairs-matching test are number of errors that participants made and therefore, higher scores reflect poorer cognitive functions. Fluid intelligence was evaluated using verbal numerical logic and reasoning-type questions. Reaction time was measured using a timed symbol matching test.

Other than with measures of cognitive tests, depressive symptoms were measured by the 4-item Patient Health Questionnaire-4 (PHQ-4) (21). For more details and information of reliabilities and validities of these cognitive measures, see **Supplementary Methods**.

### Structural Magnetic Resonance Imaging (MRI) Acquisition and Preprocessing for Structural Analyses

MRI imaging data was obtained during the third and fourth assessment visits. Images were obtained from three imaging centers equipped with identical scanners (Siemens Skyra 3T running VD13A SP4 with a Siemens 32-channel RF receive head coil, Munich, Germany).

For all preprocessing and analyses of imaging data, a computer with an Intel, Xeon, processor E5-1620 v4CPU, Windows 10 Pro Workstations OS, and 64 GB memory was used.

T1-weighted structural images were used for voxel-based morphometry analyses. We used Statistical Parametric Mapping 12 (SPM12) on MATLAB 2019b for this procedure. Images were segmented and segmented GM and WM were normalized using the diffeomorphic anatomical registration through exponentiated lie algebra (DARTEL) procedure, modulated, smoothed (8 mm full width at half maximum (FWHM), and the resultant maps representing rGMV and rWMV analyzed. For details, see **Supplementary Methods**.

We used metrics related to DTI and neurite orientation dispersion and density imaging (NODDI) (22) to measure microstructural properties of the brain. DTI can evaluate following measurements: (a) Mean diffusivity (MD): the amount of water molecule diffusion regardless of direction, (b) axial diffusivity (AD): water molecule diffusion parallel to the tract within the voxel of interest, (c) radial diffusivity (RD): the magnitude of water diffusion perpendicular to that tract, (d) fractional anisotropy: the level of anisotropy of water diffusion. NODDI can evaluate following measurements: (A) The intracellular volume fraction (ICVF): neurite compartment density, as verified by histology in animal experiments (23), (B) isotropic volume fraction (ISOVF): the extracellular free water diffusion as well as the interstitial and cerebrospinal fluids (CSF), orientation dispersion (OD) index: the spread of fibers within an intracellular compartment. By combining these metrics with information on volumetric measures, brain structural changes can be comprehensively evaluated.

The normalization of DTI and NODDI images was performed based on a previously validated protocol (24). We used SPM8 (see **Supplementary Methods** for the rationale for using SPM8) on MATLAB 2009b for this procedure. Briefly, diffusion images using the information of MD and FA, and modified DARTEL procedure which took account the FA signal distribution within WM areas (to align images and tracts within WM areas), were used to normalize all DTI and NODDI images as well as regional GM density (rGMD), regional white matte density (rWMD) and regional cerebrospinal fluid maps (rCSFD). Then, all normalized FA images were masked by a WM tissue probability (> 0.99) map mask to limit images to WM areas then smoothed (6 mm FWHM), while other DTI and NODDI images were masked by a mask of GM + WM tissue probability (sum > 0.99) map to limit images to GM and WM areas, and then smoothed (8 mm FWHM). For more details on these procedures, refer to the **Supplementary Methods**.

### Psychological and Non-Whole Brain Imaging Data Analyses

Psychological and non-whole brain imaging data were analyzed using Predictive Analysis Software, version 22.0.0 (SPSS Inc., Chicago, IL, USA; 2010). Multiple regression analyses were used to investigate the associations between BMI at the first assessment visit and changes in cognitive variables from the first assessment visit to the third assessment visit after correcting for confounding variables. The change of each measured variable from the first to the third assessment visit was used because the second assessment contained less data for psychological analyses than the third. The dependent variables for each multiple regression analysis were the change in scores for (A) reaction time, (B) fluid intelligence, and (C) depressive symptoms from the first assessment visit to the third assessment visit. The independent variables were sex, age at the first assessment visit, interval days between the first assessment visit and the third assessment visit, the number of times subjects underwent tests for this project at the time of the third assessment visit, cov1 – cov12 values at the first assessment visit, and BMI at the first assessment visit.

Cox proportional hazards models were used to examine the relationships between BMI and dementia of all causes. All-cause dementia was ascertained using hospital inpatient records and linkage to death register data. For more details, see **Supplementary Methods**. Subjects who had been diagnosed with dementia at the time of the first assessment or who self-reported dementia or cognitive impairment at the first assessment were removed from analysis. Subjects who reported dementia in the self-report but did not have a diagnosis dementia in either their hospital inpatient records or death register data were also removed from analysis. The time scale considered spanned from the time of the first assessment visit and until 28 February 2018. Covariates were sex, age at the first assessment visit, values of cov1 – cov12 at the first assessment visit, BMI at the first assessment visit, and reaction time at the first assessment visit (fluid intelligence data was not available for a majority of subjects). For these analyses, we treated BMI as a categorical variable and separated 30>x (obesity), 30≧x>25 (overweight), 25≧x>18.5 (normal), and 18.5≧x (underweight), as has been widely performed in the field.

For psychological analyses, results with a threshold of *P* < 0.05 that were corrected for false-discovery rates (FDRs) using the two-stage sharpened method (25) were considered statistically significant. This correction was applied to results of the four analyses mentioned above that treated the BMI as a continuous variable. The statistical threshold of uncorrected p < 0.05 was used for all *post-hoc* and supplemental analyses as the supplementary analyses are confirmatory.

### Imaging Data Analysis

Our study employed a voxel-by-voxel multiple regression analysis that adjusted confounding imaging variables, such as baseline-imaging measurements and tissue concentrations, at each voxel.

Statistical Parametric Mapping 5 (SPM5) and its extension software, the biological parametric mapping tool (BPM; www.fmri.wfubmc.edu) (26), on MATLAB 2008b were used for the statistical analysis of imaging data. Longitudinal whole brain multiple regression analyses were used to look for associations between BMI and post-experiment brain images (rGMV, rWMV, and DTI and NODDI images) using the data from the third assessment visit as a baseline and data from the fourth assessment visit at follow-up. This is because imaging data was available only from the third and fourth assessment visits.

In rGMV and rWMV analyses, the independent variables were sex, age at the third assessment visit, the number of interval days between the third and fourth assessment visits, values of cov1 – cov12 at the third assessment visit (except for cov1 and cov2, which refer to values at recruitment), head size ratio at the third assessment visit (calculated using UK Biobank output), BMI at the third assessment visit, and the imaging measurement at each voxel during the third assessment visit. Effects of baseline-imaging measurement (which corresponds to the third assessment visit, **Figure 1**) were corrected for on voxel-by-voxel basis using BPM. The dependent variable at each voxel was the value of the follow-up image made during the fourth assessment visit. The *p* and t values were the same when baseline outcome measurements were included as independent variables regardless of whether the outcome measurement at the follow-up assessment or the difference between the baseline and follow-up outcome measurements were used as dependent variables. The results of longitudinal analyses were thus interpreted as the associations between BMI at baseline and the changes in each imaging parameter between baseline and follow-up measurements. For DTI and NODDI images, analyses were performed in a similar manner to rGMV and rWMV analyses except that rWMD and rCSFD images of the third and fourth assessment visit (baseline and follow-up) were included as covariate images to correct for the effects of tissue probabilities of WM and CSF. The sets of covariates used in each analysis are summarized in **Table 1**.

**Table 1:** Sets of covariates and how they are used in each analysis.

	Analyses for cognitive measures	Analyses for dementia	Analyses for imaging measures
Age	〇(1^st^ visit)	〇(1^st^ visit)	〇(3^rd^ visit)
Sex	〇	〇	〇
Intervals between assessments of outcome measures	〇(those between 1^st^ and 3^rd^ visits)	×	〇(those between 3^rd^ and 4^th^ visits)
Neighborhood-level socioeconomic status	〇(1^st^ visit)	〇(1^st^ visit)	〇(1^st^ visit)
Education level	〇(1st visit)	〇(1st visit)	〇(1st visit)
Household income	〇(1^st^ visit)	〇(1^st^ visit)	〇(3^rd^ visit)
Current employment status	〇(1^st^ visit)	〇(1^st^ visit)	〇(3^rd^ visit)
Physical activity level (MET)	〇(1^st^ visit)	〇(1^st^ visit)	〇(3^rd^ visit)
Number in household	〇(1^st^ visit)	〇(1^st^ visit)	〇(3^rd^ visit)
Current tobacco smoking level	〇(1^st^ visit)	〇(1^st^ visit)	〇(3^rd^ visit)
Current alcohol drinking level (unit)	〇(1^st^ visit)	〇(1^st^ visit)	〇(3^rd^ visit)
Use of statin	〇(1^st^ visit)	〇(1^st^ visit)	〇(3^rd^ visit)
Diagnosis of diabetes	〇(1^st^ visit)	〇(1^st^ visit)	〇(3^rd^ visit)
Ethnicity	〇	〇	〇
TV viewing length	〇(1^st^ visit)	〇(1^st^ visit)	〇(3^rd^ visit)
Outcome measure at baseline	〇(1^st^ visit)	- (exclusion of dementia at baseline)	〇(3^rd^ visit, at each voxel)
Others	Number of times subjects underwent tests for this project at the time of the third assessment visit	Reaction time (1^st^ assessment)	Head size ratio (3^rd^ visit), rWMD, rCSFD (3^rd^ and 4^th^ visits, at each voxel) in DTI and NODDI analyses, except the FA analysis

For rGMV and rWMV analyses, only voxels with a signal intensity of >0.10 for all subjects were included for whole brain analyses. FA image analysis was limited to areas of WM tissue probability > 0.99 and other DTI and NODDI image analyses were limited to areas of GM+ WM tissue probability > 0.99 (see **Supplementary Methods** for a description of mask creation procedures).

Multiple comparison corrections were performed using the FDR approach (27). Areas that surpassed the extent threshold (28) based on a cluster determining threshold of P < 0.05 were corrected for FDR as described in our previous study (29). The sets of continuous voxels were treated as single clusters, as were the cases of result presentation in SPM. Adding to voxel-level corrections for multiple comparisons, cluster wise corrections for multiple comparisons were also considered to exclude the results of tiny areas among extensive areas of significance.

The descriptions in this subsection were largely reproduced from another study of ours that used the UK Biobank data (Takeuchi and Kawashima, submitted).

## Results

### Basic Baseline Data

Basic psychological data for all participants at the first assessment in this project is provided in **Supplementary Table 1**. Simple correlation coefficients of the associations between BMI and psychological variables used as covariates in following multiple regression analyses were all <0.25 in both the baseline assessment of psychological analyses and imaging analyses. These results exclude the concern of the multicollinearity in following multiple regression analyses.

### Longitudinal Psychological Analyses

For psychological data analyses, we used the data from the first and third assessment visits. The mean age of participants was 56.5 years old [standard deviation (SD): 8.0, range: 37-73] at the first assessment, with a mean interval of 3,273.9 days (SD: 642.1, range: 1,400-5,043 days) for participants who were in both assessments. After correcting for confounding variables, multiple regression analyses revealed that greater BMI at the first assessment visit was significantly associated with a greater increase in the depressive tendency, and greater increase in performance of visuospatial memory task (greater decline in number of errors before completion in the correctly ended trials), but not with change in performance of fluid intelligence tasks or in reaction time (**Table 2**). Statistical values and the number of subjects in each analysis are provided in **Table 2**.

**Table 2:** Statistical values describing associations between BMI and longitudinal changes in psychological measures (longitudinal multiple regression analyses).

Dependent variables	N	Standardized beta	T	P (uncorrected)	P (FDR)
Fluid intelligence	13409	−0.009(-0.025~0.007)	−1.074	0.283	0.283
Reaction time	39782	0.009(8.437*10^-5^~0.018)	1.978	0.049	0.064
Visuospatial memory (number of errors)	39191	-0.019(-0.027~-0.016)	-4.854	0.000001	0.000002
Depressive symptoms	38215	0.036(0.027~0.045)	7.911	2.627*10^-15^	1.051*10^-14^

### Prospective Analysis of Dementia

**Figure 2:**
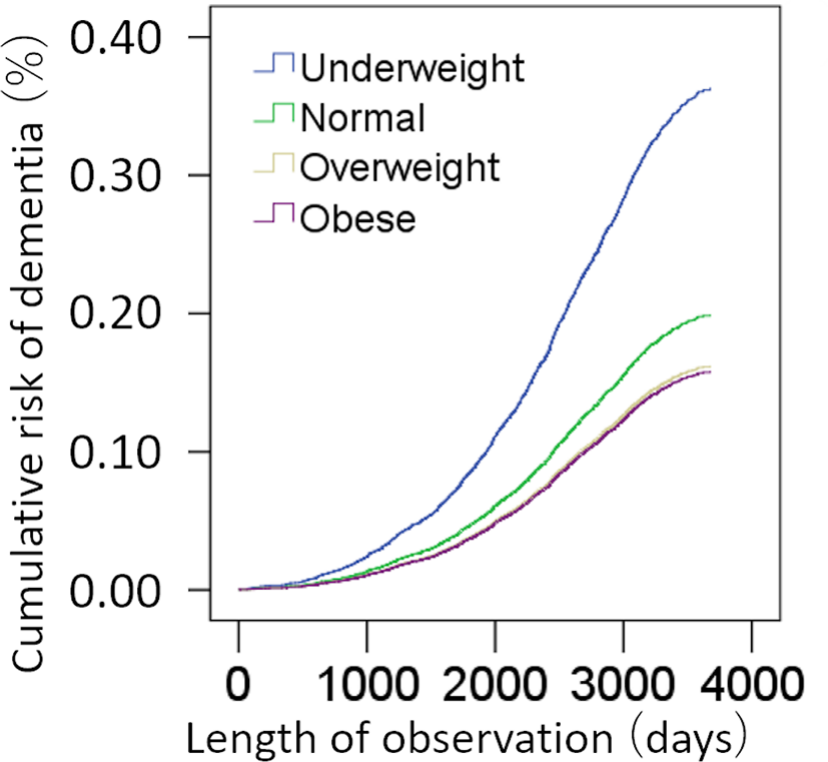
Standardized risks of incidence of dementia according to the BMI. Cox proportional hazards models were adjusted for potential confounding variables.

A total of 398,782 participants were included in this analysis. Among these, 1,593 cases of dementia were observed. Cox proportional hazard models in which BMI were divided into four categories variables [underweight (N=1994), normal (N=132014), overweight (N=170876), obese (N=94898)] revealed compared with subjects who were obese at baseline, the risk of subjects with overweight at baseline [hazard ratio (HR): 1.025, 95% confidence interval (CI): 0.904 – 1.162, p = 0.699], but subjects who were normal at baseline (HR: 1.260, 95% CI: 1.096 – 1.448, p = 0.001) and subjects who were underweight at baseline (HR: 2.296, 95% CI: 1.316 – 4.007, p = 0.003) showed significantly higher risk of incident dementia (**Figure 2**). When a continuous variable of BMI was used instead of categorical variable in this analysis, BMI was significantly and negatively associated with risk of incident dementia (p = 0.001, increasing BMI by 1 is associated with HR of 0.981 (CI:0.969-0.992)).

### Longitudinal Brain Imaging Analysis

Data from the third and fourth assessments were used for brain imaging data analysis. Participants’ mean age was 62.1 years (SD: 7.1, range 46-79) at the third assessment, mean BMI was 26.3 (Sd:4.2, Range: 13.4-50.6) and the mean interval between the third and fourth assessment visit was 822.1 days (Sd: 42.1, Range: 733-965 days) for 1253 participants (underweight:13, normal:512, overweight:517, obese:211) whose data was used in rGMV and rWMV analyses. Other characteristics of participants analyzed in imaging analyses at the third assessment were provided in **Supplementary Table 2**. The number of participants in DTI and NODDI analyses was 1241.

**Figure 3:**
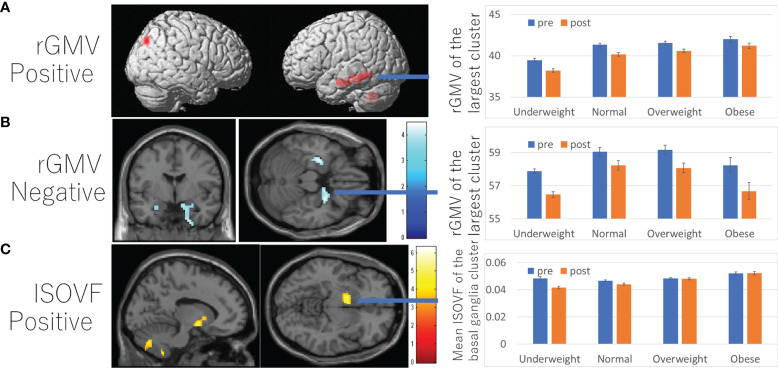
Associations between BMI and subsequent longitudinal changes in brain imaging measures. **(A)** Positive associations between BMI and rGMV change. **(B)** Negative associations between and rGMV change. **(C)** Positive associations between BMI and ISOVF change. **(A–C)** (left panels) Results are shown with a threshold of *P* < 0.05 and corrections for multiple comparisons in cluster size tests with a voxel level cluster determining threshold of *P* < 0.05 (corrected for FDR). **(A)** Regions with significant associations were overlaid on a “render” image from SPM5. **(B, C)** The findings were overlaid on a “single-subject T1” SPM5 image. The color represents the strength of the T value. (Right panels) Profiles of imaging values at pre and post scans (which correspond to the third and the fourth assessment visits) in the significant cluster of the left temporal gyrus **(A)**, right hippocampus **(B)**, and left basal ganglia areas **(C)**. For all figures, the software used to present the results was selected based on the ease of presenting the intended results. Brain images on which the results were displayed were chosen based on the default availability in the software used and whether the brain images had brain sulci.

**Figure 4:**
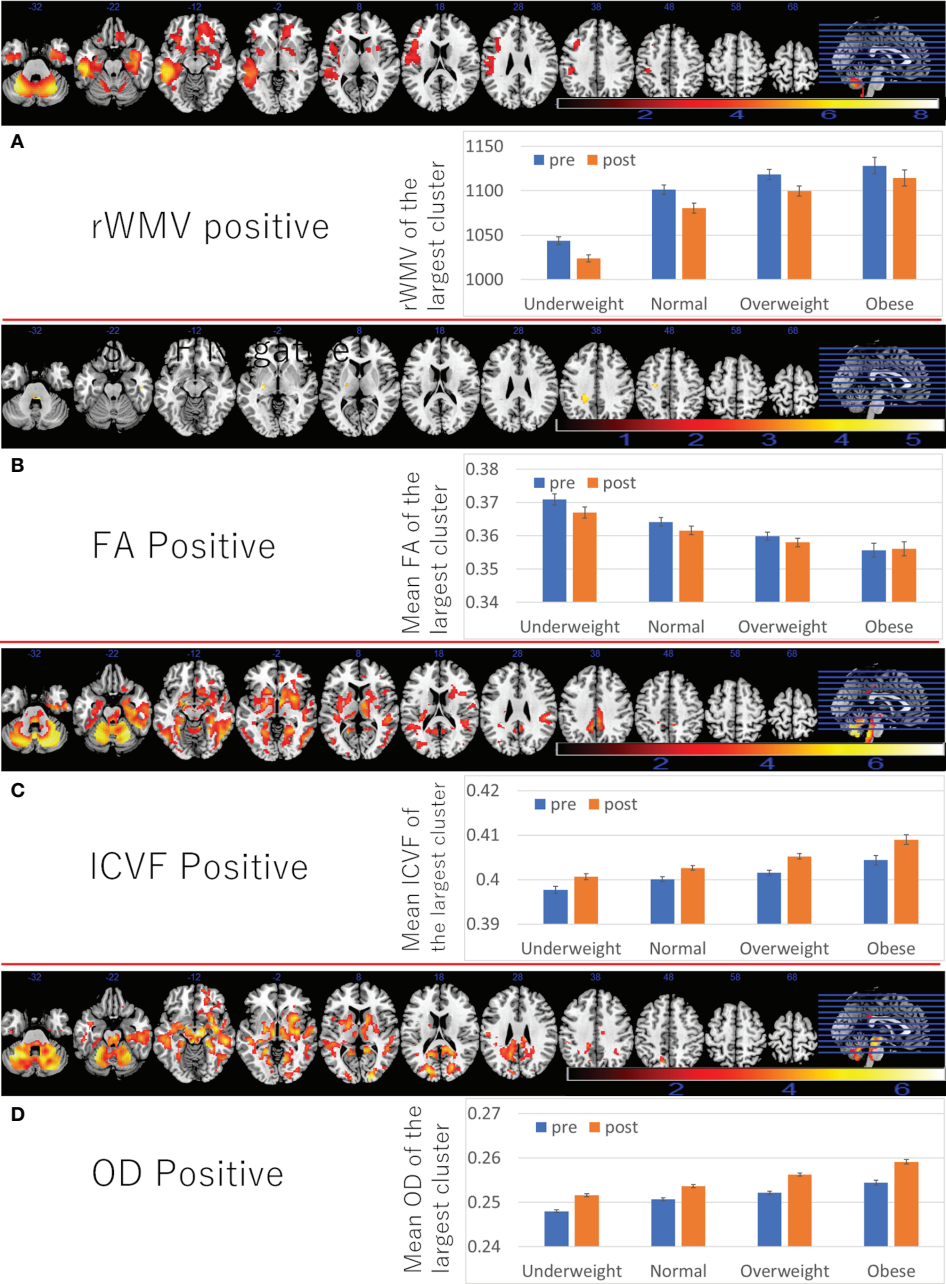
Associations between BMI and subsequent longitudinal changes in brain imaging measures. Positive associations between BMI and **(A)** rWMV change, **(B)** FA change, **(C)** ICVF change, **(D)** OD change. (Left panels) Results are shown with a threshold of *P* < 0.05 and corrections for multiple comparisons in cluster size tests with a voxel level cluster determining threshold of *P* < 0.05 (corrected for FDR). Areas of significant associations were overlaid on a “ch2bet” image using MRIcron (https://www.nitrc.org/projects/mricron) and in slices (from the left) of z = -32, -22, -12, -2, 8, 18, 28 38, 48, 58, and 68. The color represents the strength of the T value. (Right panels) Profiles of imaging values at pre and post scans (which correspond to the third and the fourth assessment visits) in the largest significant clusters of each area.

A whole brain multiple regression analysis using VBM analysis revealed significant positive associations between BMI at the third assessment and changes in rGMV from the third to the fourth assessment in the left cerebellum, left inferior/middle temporal gyrus, and right angular/occipital gyrus. It also revealed significant negative associations between BMI and rGMV change in the bilateral areas around the medial temporal lobe (**Figures 3A, B** and **Supplementary Table 3**). BMI was also positively associated with rWMV changes in the widespread areas, mainly in the WM areas around the bilateral insula, medial temporal lobes, bilateral striatum, orbitofrontal cortex, and the left lateral prefrontal and temporal lobes (**Figure 4A** and **Supplementary Table 4**).

Also, the positive associations between BMI and FA changes were found in several areas, of the left corona radiata, left internal capsule, and bilateral cerebellum (**Figure 4B** and **Supplementary Table 5**).

**Figure 5:**
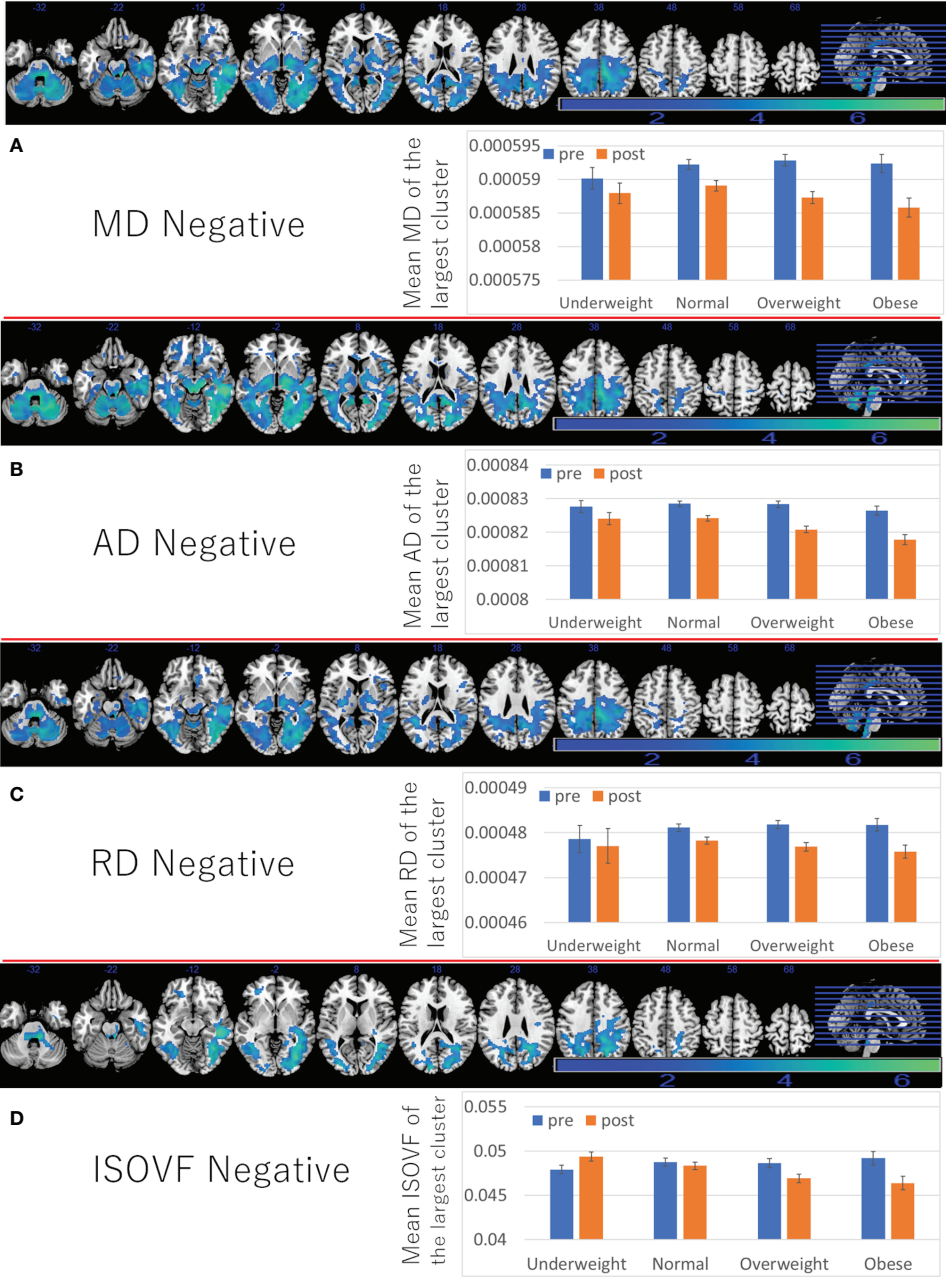
Associations between BMI and subsequent longitudinal changes in brain imaging measures. Negative associations between BMI and **(A)** MD change, **(B)** AD change, **(C)** RD change, **(D)** ISOVF change. (left panels) Results are shown with a threshold of *P* < 0.05 and corrections for multiple comparisons in cluster size tests with a voxel level cluster determining threshold of *P* < 0.05 (corrected for FDR). Areas of significant associations were overlaid on a “ch2bet” image using MRIcron (https://www.nitrc.org/projects/mricron) and in slices (from the left) of z = -32, -22, -12, -2, 8, 18, 28 38, 48, 58, and 68. The color represents the strength of the T value. (Right panels) Profiles of imaging values at pre and post scans (which correspond to the third and the fourth assessment visits) in the largest significant clusters of each area.

Further, positive associations between BMI and ICVF, OD change, as well as negative associations between BMI and changes in MD/AD/RD/ISOVF were found in the similar areas and spread widespread areas around the orbitofrontal, middle and lateral occipital, temporal and parietal lobes, basal ganglia, brain stem, and cerebellum (**Figures 4C, D**, **5C, D** and **Supplementary Tables 6–8**). However, significant associations with ICVF were mostly observed in GM areas, while the significant associations with MD/AD/RD/ISOVF were present in both GM and WM areas. There were no significant associations between BMI and ISOVF in the striatum. Instead, the positive associations between BMI and ISOVF were found in the areas of the left striatum together with the areas of bilateral cerebellum (**Supplementary Table 7** and **Figure 3C**).

Although the reasons were not clear, pre- to post average changes, which correspond to changes from the third assessment visit to the fourth assessment visit, of MD/AD/RD (decrease), ICVF (increase), ISOVF (decrease), and OD (increase) in widespread areas (**Figures 4**, **5**) were different to previously reported associations of these measures with aging (positive associations of age with MD/AD/RD, ISOVF, and negative associations of age with ICVF and OD) (16). However, cross-sectional analyses revealed mostly the same association patterns of these measures with age as those reported previously (**Supplementary Tables 5–8**). However, in some imaging measures, clusters including the brain stem often do not show clear correlation with age unlike clusters in other areas (rWMV, ISOVF) or show opposite correlation with age (FA), suggesting unique properties of this area.

### Effects of Diabetes

We included diagnosis of diabetes at baseline as a covariate, as has been done elsewhere. However, since diabetes is partly assumed to be a consequence of obesity (rather than a confounding factor), we evaluated effects of this covariate and how the statistical values in each analysis changes when this diagnosis of diabetes at baseline is removed. For this evaluation, in imaging analyses, we used total imaging variables (e.g., total GMV).

Diagnosis of diabetes at baseline was significantly associated with greater reaction time, depressive tendencies, and risk of dementia in each main analysis, but not with fluid intelligence and total and mean imaging variables. Removing this covariate from the analyses made the associations between BMI and reaction time stronger (beta value changed from 0.009, uncorrected to 0.012), but remained the status of significance of associations between BMI and other measures. For details, see Supplemental Results and **Supplementary Table 9, 10**.

We then divided the subjects into four groups according to the presence of overweight/obesity (BMI > 25 or not) and presence of diabetes and compared the outcome differences by analyses of covariance. The independent and dependent variables coincide with those of analyses for the effects of diabetes as described above, except that we excluded the variables BMI and diabetes and added a variable of four categories [based on the existence of overweight/obesity (BMI >25) and diabetes instead. The effects of diabetes and overweight/obesity are generally similar to those of BMI and diabetes in the results of the analyses for the effect of BMI alone and analyses for the effect of diabetes alone. The effect of overweight/obesity lost significance in some brain imaging analyses, probably because of the information loss due to dichotomous classification. In these psychological index and brain imaging analyses, the non-overweight/obese diabetic group did not differ significantly from the other groups, probably due to the small sample size. In the analysis of dementia risk, compared to the non- overweight/obese non-diabetic group (reference), the non-overweight/obese diabetic group showed significantly higher risk (HR: 1.955, 95% CI: 1.345–2.843, p = 4.49 × 10^−4^), the obese non-overweight/obese group showed significantly lower risk (HR: 0.800, 95% CI: 0.713–0.898, p = 1.44 × 10^−4^), and the overweight/obese diabetic group showed significantly higher risk (HR: 1.517, 95% CI: 1.269–1.814, p = 5.0 × 10^−6^). These results may indicate the increased risk of dementia based on diabetic status outweigh the decreased risk of dementia based on overweight/obese status (BMI > 25). For adjusted group values, incidence of dementia, and statistical values in each analysis, see **Supplementary Table 11**.

### Effects of Hyperlipidemia

We also evaluated how the existence of hyperlipidemia at baseline impacts the outcome measures when added as a covariate in addition to all the covariates in the main analyses, and how that addition affects the significance of the effects of BMI on outcome measures. Since there was no available data on blood lipid levels at baseline, we used the HES record of diagnosis and self-reported data to define existing hyperlipidemia. As in the supplemental analyses of diabetes, in these supplemental imaging analyses, we used total imaging variables (e.g., total GMV).

Existence of hyperlipidemia at baseline was significantly associated with a significant increase of depressive tendencies (beta = 0.024 (CI:0.013–0.035), t = 4.445, p = 9.0 * 10^−6^) and of risk of dementia (HR: 1.256, 95% CI: 1.102 – 1.431, p = 0.001) in each supplemental analysis, but did not show a tendency of associations with other variables. Adding this covariate (existence of hyperlipidemia) to the analyses affected the significance of each of associations between BMI and outcome measures little (compared with analyses without the covariate of existence of hyperlipidemia). For details, see **Supplementary Methods**, and **Supplementary Table 12**.

In these analyses, the effect of hyperlipidemia at baseline on the longitudinal change of depressive tendencies was similar to the effects of higher BMI but its effect on the risk of dementia was the opposite to those of greater BMI, and unrelated to other psychological and imaging correlates of BMI, suggesting differing effects of BMI and hyperlipidemia.

### Evaluation of the Impacts of Excluding Subjects With Comorbidities on the Effects of BMI on Outcome Measures

Next, we conducted two types of analyses excluding subjects with key comorbidities. One type involved analyses excluding subjects only subjects with cancers and other serious medical conditions. The other involved analyses excluding subjects with major comorbidities (11 types of comorbidities: diabetes, hyperlipidemia, angina, heart attack, high blood pressure, stroke, blood clot in the leg, blood clot in the young, emphysema/chronic bronchitis, cancer, other serious medical conditions/disabilities). The former analyses were conducted to evaluate the effects of BMI after excluding subjects with a possible source of underweight. The latter to observe the effects of pure among healthy subjects without complications of obesity, or the possible cause of underweight.

In analyses removing subjects with cancers and other serious medical conditions/disabilities, the effects of BMI mostly did not change, however, except on total WMV which became insignificant. Further, the negative effects of BMI on total ISOVF became significant. As for the results of rWMV, since adjustment of these variables of cancers and serious medical condition/disability (instead of subject removal) did not erase the significance of the association between BMI and greater rWMV preservation. This change may suggest that the effects of BMI on total rWMV may be affected by participants with comorbidities such as cancers and other serious medical conditions (e.g., with one of these conditions, higher BMI tends to lead to greater rWMV preservation). In case of ISOVF, the results may suggest, that without these comorbidities, a higher BMI is associated with a lower increase in ISOVF (interstitial fluids and CSF).

In analyses removing subjects with eleven types of comorbidities, the effects of BMI on psychological measures mostly unchanged, though the effects of BMI on visuospatial performance slightly decreased and became marginally significant. In case of imaging analyses, for the WMV and ISOVF results, the same pattern of change was observed as in the analysis excluding cancer patients. In addition, in this analysis removing eleven types of comorbidities, the positive effects of BMI on total rGMV became significant. These changes may reflect that in absence of comorbidities that harm the brain such as stroke, higher BMI is associated with greater GMV preservation.

Full details of methods and statistical values are shown in **Supplementary Methods**, and **Supplementary Table 13**.

## Discussion

The present study revealed novel associations between BMI and subsequent changes in microstructural properties of brain measured by DTI and NODDI as well as changes in rGMV and rWMV with voxel-by-voxel analyses in the elderly. We revealed positive associations between BMI and subsequent changes in rGMV in multiple areas, rWMV changes in widespread WM tracts, FA changes in several tracts, and ICVF and ODI in widespread areas, and ISOVF in a few areas. We also demonstrated negative associations between BMI and subsequent changes in rGMV in the bilateral medial temporal lobe areas, and MD/AD/RD/ISOVF in widespread areas. Higher BMI was not significantly associated with greater cognitive decline, unlike previous studies after adjusting for a wide range of potential confounding variables, greater increase of depressive tendencies, and lower risk of onset of dementia, consistent with previous studies. We also newly showed higher was significantly associated with greater relative increase (retention) of performance of visuospatial memory task.

Mostly consistent with our first hypothesis, lower rather than higher BMI was associated with subsequent changes in brain structures, similar to those observed in aging and dementia. Specifically, BMI was positively correlated with subsequent changes in rGMV in multiple areas, rWMV changes in widespread WM tracts, FA changes in several tracts, and ICVF in widespread areas, and was negatively correlated with changes in MD/AD/RD/ISOVF in widespread areas. These associations were mostly opposite to the previously reported associations between age or dementia and these imaging indices (16, 30, 31). A cross-sectional analysis of the present study did indeed reveal that higher age was associated with lower rGMV, rWMV, FA, ICVF, and greater MD/AD/RD/ISOVF (**Supplementary Tables 3–8**). Therefore, our imaging indices reveal that neural changes in those with higher BMI is mostly contrary to what is observed in higher age and dementia. As described in the Introduction, a previous study showed that obesity is associated with higher relative WMV increase in ROI analyses (12), and another small sample study showed that obese subjects have enlarged WMV and diet in these subjects led to WMV reduction (however, the changes were not compared with the control group) (32). The present rWMV analyses, as well as the longitudinal decline in hippocampal volume, are consistent with these studies (12, 13) and advance these previous ROI-based findings of GMV and WMV with larger sample sizes and voxel-by-voxel analyses. In addition, we provided new findings of longitudinal DTI and NODDI analyses related to BMI. The possible micro-level mechanisms behind neuroimaging findings, and why lower BMI in late life seems to precede the accelerated neural changes observed in aging and dementia are unclear and we can only speculate, and such discussions were provided in **Supplementary Discussion**.

Unlike other imaging indices, the positive associations between higher BMI and subsequent OD change were in the same direction as cross-sectional associations between age and OD in the same areas. OD is supposed to reflects angular variation of neurites (33) and is therefore, lower in low crossing fiber areas such as the corpus callosum. Previous studies have reported that the association between age and OD differ substantially depending on brain areas, some areas show age-related increase, while others show age-related decrease and yet other regions do not show clear associations with age (16). In an animal model of Alzheimer’s disease, amyloid beta accumulation was associated with increase of OD (34). Thus, one possibility of the mechanisms behind characteristic patterns of OD is high OD reflects multiple neural mechanisms and physiological factors such as relative dendrite increases caused by higher BMI, as well as pathological changes such as accumulation of amyloid beta or misalignment of neural tracts caused by aging. But these are speculations and further investigation is required to confirm these possibilities.

Interestingly, higher BMI was associated with greater rGMV decline in the bilateral hippocampus, which is consistent with findings from ROI analyses in a previous study (13). Reduction in volumetric measures in the amygdala and the hippocampus is robustly seen in major depressive disorder (35, 36). Therefore, the present association between BMI and subsequent specific change of this area may be related to the association between higher BMI and subsequent increase of depressive tendency. The reason why the associations between BMI and these neurocognitive changes are seen is not clear. But we provided possible mechanisms behind these associations in **Supplementary Discussion**.

Unlike widespread areas of significant findings of ISOVF, higher BMI was associated with subsequent relative increase of ISOVF in the left striatum. Previous psychological studies suggest that higher BMI in normal to mildly obese subjects is associated with increased sensitivity to reward, suggesting greater dopamine availability or higher sensitivity of the dopamine pathway (37). Our cross-sectional study showed lower MD in the dopaminergic system and higher BMI and dopamine receptor density, suggesting facilitation of reward circuits in mildly obese subjects (15). Greater relative increase of ISOVF in the striatum may reflect some consequences of higher BMI, such as increase of cerebral blood flow. However, cerebral blood flow at rest data was not available in this study and future studies need to investigate this issue.

The present study did not find an association between higher BMI and subsequent cognitive decline, rather higher BMI was associated with subsequent greater retention in visuospatial memory performance. At first glance, the results of the present study might not appear to be consistent with some previous research of a much smaller sample size (>10x) than the present study (8). This superficial discrepancy may be due to critical confounding variables such as education level and length of TV viewing. After adjusting for a wide range of representative potential confounding variables, we found that higher BMI was associated with subsequent greater retention in visuospatial memory performance and was not significantly associated with greater decline in fluid intelligence, although there was a trend for an association with longer reaction time. In the present dataset, our additional analyses revealed other than basic covariates (age, sex, and interval), removal of education levels, and length of TV viewing substantially strengthen the associations between higher BMI and greater decline in fluid intelligence (though, even in this case, higher BMI was still significantly associated with greater subsequent retention in visuospatial memory performance). When the education level is removed from the covariate, statistical values of associations between BMI and change in performance of fluid intelligence changed from p = 0.283 (uncorrected), beta = -0.009 to p = 0.057 (uncorrected), beta = -0.015, and further removal of length of TV viewing changed statistical values to p = 0.001 (uncorrected), beta = -0.027. In previous studies, even when the associations between BMI and subsequent cognitive decline are shown, when the model included education levels as a covariate, associations become only marginal significant in a few measures (8). Therefore, although our findings might not appear consistent with previous research, the pattern of significance of the associations between higher BMI and greater decline of higher order cognitive functions without correcting for education and TV viewing as well as the substantially weakened associations after adjusting for education level, is actually congruent with said previous study (8). And length of TV viewing is not included as a covariate in previous studies that have shown the associations between BMI and subsequent cognitive decline (8). More recent studies including the study on UK Biobank data, support an association between prolonged TV viewing and subsequent greater cognitive decline (38–40). Therefore, using a sample size ten time larger than previous studies, we showed previously reported associations between higher BMI and subsequent cognitive decline are at best elusive and could be due to effects of unadjusted length of TV viewing, rather than BMI itself. Similarly, removing effects of education in the analysis of reaction time changed statistical values of associations between BMI and reaction time change from p = 0.048(uncorrected), beta = 0.009 to p = 0.016 (uncorrected), beta = 0.011.

In the UK Biobank cohort, the mean age of participants was 56.5 years old (SD: 8.0) at the first assessment, with a mean interval (between the first and the third assessment) of 3,273.9 days (SD: 642.1) for participants present in both assessments when our data for psychological analyses were obtained. In addition, the follow-up period for prospective analyses of risk of dementia was about 9 years in average. Age-related cognitive decline in several domains as well as volumetric reduction of brain GM and WM begin around or until this age (mid 50’s) (41, 42), and the incidence ratio of dementia is still relatively low before the age of 70 (43). Therefore, certain sensitivity in the analyses of dementia may be lost. However, the large sample size compensates this limitation. Furthermore, numerous representative prospective studies of dementia have been performed using data from the UK Biobank (e.g.,^44^), and our abovementioned results were also significant. Some UK Biobank studies conduct their analyses by restricting baseline age to≧60 (e.g.,^44^). Even when we conducted the analyses among a sample of subjects with baseline age >60 years old, the significance of the main prospective analyses of dementia was not affected (P = 0.008, in the analysis treating BMI as a continuous variable). But future studies are required to observed if there are differing patterns in a longer follow-up period.

This study has a few limitations. First, this study is not an intervention study. While we corrected for a wide range of potential confounding variables, the effects of factors that could not be adjusted remain. Factors that drove individuals into greater or smaller BMI, rather than BMI itself may play a key role in forming the observed associations between BMI and neurocognitive variables. Particularly, some suggest that lower BMI may be a pre-clinical sign of dementia, though other studies effectively ruled out such possibilities (6). In our multiple regression analyses, although we adjusted the baseline variable of outcome measures in each analysis, the possibility that low BMI is an expression of certain underlying progressive neurological states cannot be ruled out. Future intervention studies may be required to consider such possibilities. In addition, due to the design of UK Biobank, we used the 1^st^–3^rd^ visit for psychological analyses (this combination had the biggest sample size) and 3^rd^–4^th^ visit for imaging analyses (imaging data was only available in these visits). Therefore, the age of cognitive and imaging changes overlapped but not coincided and subjects that were analyzed in psychological and imaging are different, and this fact made combined analyses of psychological and imaging data difficult. Another limitation is that in the UK Biobank cohort, 94.6% of participants are of white ethnicity and rest of ethnicities are highly divided. Therefore, it is difficult to estimate the effects of the interaction between certain ethnic differences and BMI. Particularly, it is known that BMI classification as obese differs between Asian (BMI ≧ 25) and Western standards (BMI ≧ 30) (45). Thus, future studies should investigate if the effects of BMI on neurocognitive changes in the elderly differ in Asians.

In this current study, we advanced understanding of the effects of BMI by showing that higher BMI was mostly associated with relative structural changes opposite to those seen in aging processes and dementia. Previously, there were controversies regarding whether high BMI in the elderly is associated with the decreased risk of incident dementia, and increased risk of subsequent cognitive decline. Higher BMI was associated with greater retention in visuospatial memory performance, but not associated with subsequent cognitive decline of fluid intelligence and reaction time after adjustment of potential confounders, but the insignificance of fluid intelligence depends on the adjustment of length of TV viewing and education, and insignificance of reaction time depends on depends on the adjustment of education and diagnosis of diabetes at baseline. Higher BMI was also associated with lower risk of dementia, but its association was substantially weakened when diagnosis of diabetes was not adjusted at baseline. Voxel-by-voxel longitudinal analyses revealed regional increases (lateral temporal, parietal areas, cerebellum) and decreases (medial temporal areas) of rGMV. Overall, VBM, DTI and NODDI analyses revealed results mostly consistent with neuroprotective effects of higher BMI with the exception of OD and some areas of ISOVF findings. Most of the significant longitudinal associations of higher BMI were opposite to those observed in higher age and dementia as was the case of the association between BMI and subsequent risk of sarcopenia (46). Future epidemiological studies should consider separating effects of higher BMI itself, potential confounders, as well as obesity related diseases such as diabetes. Further attention should be paid to the phenomenon that lower BMI in older adults precedes the accelerated neural changes observed in the elderly.

## Data Availability Statement

The datasets presented in this article are not readily available because the data is accessible through the request to UK Biobank. Requests to access the datasets should be directed to https://bbams.ndph.ox.ac.uk/ams/researcher_home.jsp.

## Ethics Statement

The studies involving human participants were reviewed and approved by the North-West Multi-center Research Ethics Committee. The patients/participants provided their written informed consent to participate in this study.

## Author Contributions

HT conceptualized the study, preprocessed, analyzed the data and wrote the manuscript. RK plays a key role in obtaining the relevant funding and supervised the study. All authors contributed to the article and approved the submitted version.

## Funding

The UK Biobank was supported by the Wellcome Trust, Medical Research Council, Department of Health, Scottish government, and Northwest Regional Development Agency. It has also had funding from the Welsh Assembly government and British Heart Foundation. This particular study of authors is supported by JST/RISTEX, JST/CREST. The research was designed, conducted, analyzed, and interpreted by the authors entirely independently of the funding sources.

## Conflict of Interest

The authors declare that the research was conducted in the absence of any commercial or financial relationships that could be construed as a potential conflict of interest.

## Publisher’s Note

All claims expressed in this article are solely those of the authors and do not necessarily represent those of their affiliated organizations, or those of the publisher, the editors and the reviewers. Any product that may be evaluated in this article, or claim that may be made by its manufacturer, is not guaranteed or endorsed by the publisher.
