# Supplementary material for: Effects of Body Mass Index on Brain Structures in the Elderly: Longitudinal Analyses
Source: Front Endocrinol (Lausanne). 2022 Jun 3;13:824661. doi: 10.3389/fendo.2022.824661 (PMC9204255; doi:10.3389/fendo.2022.824661)
Supplement: Supplementary file 1 [file DataSheet_1.docx]

**Supplementary online material**

**Supplemental Methods.**

**Additional information on subjects**

Adults aged 40–69 years old who were registered with the National Health Service (NHS) and lived within 25 miles of the study's evaluation sites were invited by email to take part in the UK Biobank. No exclusion criteria were applied for this recruitment.

**Details on sociodemographic and lifestyle measures**

The following variables were calculated and used in this study.

(cov1) Neighborhood-level socioeconomic status was measured by the Townsend index of material deprivation ([Townsend, 1987](#_ENREF_30)). Status was calculated based on the home postcode of the subject and represents a composite index of four postcode-level socioeconomic status variables: household overcrowding, unemployment, non-home ownership, and non-car ownership). A higher score implies a lower socioeconomic status. This value was obtained at recruitment and used for all analyses.

(cov2) The education level was based on self-reported data. Education level categories of participant choices were transformed into numerical values as previously described ([Okbay et al., 2016](#_ENREF_20)): “College or University degree” = 20 years; “A levels/AS levels or equivalent” = 13 years; “O levels/GCSEs or equivalent” = 10 years; “CSEs or equivalent” = 10 years; “NVQ or HND or HNC or equivalent“ = 19 years; “Other professional qualifications e.g.,: nursing, teaching” = 15 years; “None of the above” = 7 years; “Prefer not to answer” = missing. This value was obtained at recruitment and used for all analyses.

(cov3) The household income was the self-reported total income (before taxes) received by the subject's household. The available choices were <£18,000, £18,000 to £30,999, £100,000, £31,000 to £51,999, £52,000 to £100,000, >£100,000, do not know, and prefer not to answer. We converted these choices into ordinal variables of 1–5 (>£100,000 = 5) ([Shen et al., 2018](#_ENREF_24)) after excluding answers of do not know and prefer not to answer, as done elsewhere.

(cov4) Current employment status was used to describe the occupation information of the participants. The responses to the variable were: “In paid employment or self-employed,” “Retired,” “Looking after home and/or family,” “Unable to work because of sickness or disability,” “Unemployed,” “Doing unpaid or voluntary work,” “Full or part-time student,” and “None of the above.” Multiple responses were allowed. Responses were classified as either “In paid employment or self-employed” or not.

(cov5) Physical activity level was calculated from the recorded items from the International Physical Activity Questionnaire short form and was converted into a single measure of total physical activity in metabolic equivalent of task hours. For more details, see the previous study ([Cullen et al., 2018](#_ENREF_9)).

(cov6) Number of people in their household (including institutions such as care homes) was self-reported. Answers were assigned one of four variables: 1 (single person), 2 (two people), 3 (three people), and 4 (four or more people) as has been done elsewhere ([Sarkar et al., 2008](#_ENREF_22)).

(cov7) Participants were asked about the current tobacco smoking status. Possible answers were 1 (No), 2 (Only occasionally), and 3 (Yes, on most or all days). Responses of “prefer not to answer” were excluded.

(cov8) The current alcohol intake level was calculated as previously described ([Howe et al., 2019](#_ENREF_13)). Study participants were asked to describe their current drinking status (never, previous, current, prefer not to say), and estimate their current alcohol intake frequency (daily or almost daily, three or four times a week, once or twice a week, one to three times a month, special occasions only, never, prefer not to say). Individuals reporting a current intake frequency of at least once or twice a week were asked to estimate their average weekly intake of a range of different alcoholic beverages (red wine, white wine, champagne, beer, cider, spirits, fortified wine). From these variables, we calculated an average intake of alcoholic units per week. This was derived by combining the self-reported estimated intake of the different alcoholic beverages across the five drink types. This calculation used the following measurement units for each of the five alcoholic drink types: measures for spirits, glasses for wines and pints for beer/cider, were estimated to be equivalent to 1, 2 and 2.5 units respectively. Individuals reporting current intake frequency of “one to three times a month,” “special occasions only,” or “never,” were assumed to have a weekly alcohol consumption volume of 0.

(cov9) Participants reported history of statin use was collected by using a touchscreen questionnaire at each assessment visit. And the dichotomized variable (subjects who reported the use and those who did not) was generated based on this answer.

(cov10) Participants were asked about the existence of doctor diagnosis of diabetes. And the dichotomized variable (subjects who reported yes to this question, and those who reported no to this answer) was generated based on this answer. The UK Biobank data do not specify the types of diabetes.

(cov11) Ethnicity was self-reported, and possible answers were white or other.

(cov12) Time spent watching TV was assessed by the following question: “In a typical day, how many hours do you spend watching TV?”. Answers were: “less than an hour a day” or any integer value between 0 and 24, with an answer of “less than an hour a day” regarded as 0 hours. Responses of more than 6 hours were treated as 6 hours in the analysis in line with those of recent representative studies of the effects of length of TV viewing ([Hamer et al., 2017](#_ENREF_12);[Celis-Morales et al., 2018](#_ENREF_5)).

**Details of cognitive measures**

We used data from tests that were administered to a large portion of subjects. Reasoning, or fluid intelligence, was evaluated using 13 verbal numerical logic and reasoning-type questions with a 2-minute time limit and a maximum score of 13. The Cronbach alpha coefficient for each item was 0.62.

Reaction time was measured using a timed symbol matching test. In this test, for each trial, two cards with symbols were shown and participants had to press a button as fast as possible if the two cards had matching symbols. The score for this task was the mean response time across trials containing matching pairs. The Cronbach alpha coefficient for this task was 0.85.

Depressive symptoms were measured by the 4-item Patient Health Questionnaire-4 (PHQ-4) ([Batty et al., 2016](#_ENREF_3)), which was administered at all four patients visits to assessment centers. This measurement has an area under the curve of 0.79 for its correlation with a depression diagnosis ([Khubchandani et al., 2016](#_ENREF_16)). For other information on the reliability and validity of this measurement technique, see ([Khubchandani et al., 2016](#_ENREF_16)).

**Ascertainment of dementia**

For the ascertainment of dementia of all causes, we followed methods established in a previous study ([Lourida et al., 2019](#_ENREF_17)), with descriptions in this subsection mostly being reproduced from this previous study. All-cause dementia was determined based on hospital inpatient records containing data on admissions and diagnoses obtained from the Hospital Episode Statistics for England, Scottish Morbidity Record data for Scotland, and the Patient Episode Database for Wales. Additional cases were identified through death register data provided by the NHS Digital for England and Wales and the Information and Statistics Division for Scotland. Diagnoses were recorded using the International Classification of Diseases (ICD) coding system. Participants with dementia were identified as having a primary/secondary diagnosis (hospital records) or underlying/contributory cause of death (death register) using ICD-9 and ICD-10 codes for Alzheimer disease and other dementia classifications.

**Details of structural MRI acquisition and preprocessing for volumetric analyses**

For the UK Biobank study cohort, MRI imaging data was obtained for the third and fourth assessment visits. T1 weighted structural images were obtained from 3 imaging centers equipped with identical scanners (Siemens Skyra 3T running VD13A SP4 with a Siemens 32-channel RF receive head coil, Munich, Germany).

Structural images were acquired that contained straight sagittal orientations with a resolution of 1 × 1 × 1 mm and a field of view of 208 × 256 × 256, were taken over a duration of 5 minutes, and that contained 1-mm isotropic resolution generated using a 3-dimensional magnetization-prepared rapid-acquisition gradient echo.

For segmentation processes, outputs from the standard biobank processing pipeline involving FSL were used. Details of MRI protocols and segmentation are provided elsewhere ([Miller et al., 2016](#_ENREF_19);[Alfaro-Almagro et al., 2018](#_ENREF_1)).

Normalization processes were performed using Statistical Parametric Mapping software (SPM12; Wellcome Department of Cognitive Neurology, London, UK) implemented in MATLAB (MathWorks Inc., Natick, MA, USA). First, using a segmentation algorithm implemented in SPM12, T1-weighted structural images of each scan were segmented, resulting in diffeomorphic anatomical registration through exponentiated lie algebra (DARTEL) import images. White matter segment images were generated through the standard biobank processing pipeline using FSL and were co-registered and resliced with DARTEL import images of white matter segments. Using the same parameters, gray matter segment images generated through the standard biobank processing pipeline using FSL were co-registered and resliced.

Using generated images, the DARTEL registration process was performed using SPM12 and both gray matter and white matter segmentation maps generated through the standard biobank processing pipeline using FSL were normalized to the Montreal Neurological Institute (MNI) space to give images with 3 × 3 × 3 mm^3^ voxels. The DARTEL template was then created using imaging data from the 250 baseline experiment images of subjects from the third assessment visit, as well as 250 follow-up images from the fourth assessment visit of different subjects. These 500 subjects were chosen based on the participants’ data ID numbers in the projects (participants of the first 500 IDs of ones who had both baseline and follow-up images). In each UK Biobank project, subjects’ IDs were randomly assigned, thus small ID numbers do not mean the data of the IDs was obtained earlier or participants are younger. Next, using the existing template, DARTEL procedures were performed for all images. In addition, we performed a volume change correction ([Ashburner and Friston, 2000](#_ENREF_2)). rGMV and rWMV images were then smoothed by convolving them with an isotropic Gaussian kernel of 8 mm full width at half maximum.

These processes were performed because although we used DARTEL procedures for precise registration processes, the segmentation quality of the standard biobank processing pipeline using FSL was better than that of SPM12’s new segmentation method and CAT 12.

**Details of diffusion MRI acquisition and preprocessing**

We used DTI and NODDI measurements released by the UK Biobank Imaging Study, including non-normalized FA, MD, AD, RD, ISOVF, ICVF, and OD maps. Details of the dataset can be found in the protocol documentation (<https://biobank.ctsu.ox.ac.uk/crystal/docs/brain_mri.pdf>) and in a previous study ([Miller et al., 2016](#_ENREF_19)). The key elements from these documents are described below.

Diffusion data were acquired using two b-values (b = 1,000 and 2,000 s/mm^2^) with a resolution of 2 × 2 × 2 mm and a field of view of 104 × 104 × 72 over a duration of 7 minutes, with a multiband acceleration factor of 3 in which three slices are acquired simultaneously. For each diffusion-weighted shell, 50 distinct diffusion-encoding directions were acquired covering 100 distinct directions over two b-values. Both diffusion tensor and NODDI models were fit voxel-wise and the image-derived phenotypes of various model outputs extracted from a set of white matter tracts. Tensor fits utilizing the b = 1000 s/mm^2^ data were used to produce maps including MD, FA, RD, and AD. The NODDI model was fit using the Accelerated Microstructure Imaging via Convex Optimization tool ([Daducci et al., 2015](#_ENREF_10)) with outputs including ISOVF, ICVF, and OD.

Preprocessing and analysis of diffusion data were performed using SPM8 implemented in MATLAB. The following method descriptions have been largely reproduced from our previous study ([Takeuchi et al., 2013](#_ENREF_29);[Takeuchi et al., 2016](#_ENREF_26)). Using the b = 0 image, DTI and NODDI images were linearly aligned to the skull-stripped T2 image template of SPM8 to assist with the following procedures. When this registration processes did not proceed as expected, images were manually aligned to the template image.

A previously validated two-step segmentation algorithm for diffusion images and a previously validated DARTEL-based registration process were normalized ([Takeuchi et al., 2013](#_ENREF_29)) in a process involving FA signal distribution within white matter areas. All images, including segments of gray matter (regional gray matter density (rGMD) map], white matter (regional white matter density (rWMD) map], cerebrospinal fluid (CSF) (regional CSF density (rCSFD) map] of diffusion images, were normalized. The voxel size of these normalized images was 2 × 2 × 2 mm^3^. In these normalization processes, the template for the DARTEL process was created from 125 baseline (3^rd^ assessment visit) images and 125 follow-up (4^th^ assessment visit) images of different subjects.

The details of these procedures, which have also been described in our previous study ([Takeuchi et al., 2013](#_ENREF_29)), are as follows. Using the new segmentation algorithm implemented in SPM8, FA images of each individual subject were segmented into six tissues (first new segmentation). The default parameters and tissue probability maps were used for this process, except that affine regularization was performed using the International Consortium for Brain Mapping template for European brains and the sampling distance (approximate distance between sampled points when estimating the model parameters) was 2 mm. We then synthesized the FA image and MD map. In the synthesized image, the area with a WM tissue probability of >0.5 in the abovementioned new segmentation process was the FA image multiplied by −1 (hence, the synthesized image shows very clear contrast between WM and other tissues); the remaining area is the MD map (for details of this procedure, see below). The synthesized image from each individual was then segmented using the new segmentation algorithm implemented in SPM8 with the same parameters as above (second new segmentation). This two-step segmentation process was adopted because the FA image has a relatively clear contrast between GM and WM, as well as between WM and CSF, and the first new segmentation step can segment WM from other tissues. On the other hand, MD map has clear contrast between GM and CSF and the second new segmentation can segment GM. Since the MD map alone lacks clear contrast between WM and GM, we must use a synthesized image (and the two-step segmentation process).

We then performed a DARTEL registration process in SPM8. We used the DARTEL import image of the GM tissue probability map produced in the second new segmentation process as the GM input for the DARTEL process. The WM input for the DARTEL process was created as follows. First, the raw FA image was multiplied by the WM tissue probability map from the second new segmentation process within areas having WM probabilities of >0.5 (signals from other areas were set to 0). Next, the FA image * WM tissue probability map was co-registered and resliced based on the DARTEL import WM tissue probability image from the second segmentation, which created the DARTEL import image. The DARTEL template was created using imaging data from 125 baseline (3^rd^ assessment visit) experimental images of subjects and 125 follow-up (4^th^ assessment visit) experimental images of different subjects. These 250 subjects were chosen based on the participants’ data ID numbers in the projects (participants of the first 250 IDs of ones who had both baseline and follow-up images). In each UK Biobank project, subjects’ IDs were randomly assigned, thus small ID numbers do not mean the data of the IDs was obtained earlier or participants are younger. Next, using the existing template, DARTEL procedures were performed for all images. The parameters for these procedures were changed as follows to improve accuracy. The number of Gauss–Newton iterations performed within each outer iteration was set to 10 and, in each outer iteration, we used 8-fold more timepoints to solve partial differential equations than the default values. The number of cycles used by the full multi-grid matrix solver was set to 8. The number of relaxation iterations performed in each multi-grid cycle was also set to 8. The resultant synthesized images were spatially normalized to MNI space. Using these parameters, the raw FA, raw MD, rGMD, rWMD, and rCSFD maps from the abovementioned second new segmentation process were normalized to give images with 1.5 × 1.5 × 1.5 mm^3^ voxels. The FA image * WM tissue probability map was used in DARTEL procedures because it includes different signal intensities within WM tissues and because the normalization procedure can take advantage of the intensity differences to adjust the image to the template from the perspective of the outer edge of the tissue and within the WM tissue. No modulation was performed in the normalization procedure.

We next created averages from the average images of normalized WM segmentation images (rWMD) of 250 images from which the DARTEL template was created, as described above, from the mask image consisting of voxels with a WM signal intensity >0.99. We then applied this mask image to the following normalized images (FA/rGMD/rWMD/rCSFD), therefore retaining from normalized images only areas that are highly likely to represent white matter. These images were then smoothed (6 mm full-width half-maximum) and carried through to second-level analyses of FA.

We averaged normalized GM segmentation images (rGMD) and normalized WM segmentation images (rWMD) of 250 images from which the DARTEL template was created, as described above, from the mask image consisting of voxels with a GM signal intensity + WM signal intensity >0.99. We applied this mask image to the following normalized images (MD/AD/RD/ ICVF/ISOVF/OD/rGMD/rWMD/rCSFD), therefore retaining only areas that are highly likely to represent gray matter or white matter. These images were then smoothed (8 mm full-width half-maximum) and carried through to second-level analyses of MD/AD/RD/ ICVF/ISOVF/OD.

***Rationale for SPM8 use in the preprocessing of DTI data***

Concerning preprocessing, we made use of SPM8 in the preprocessing of diffusion images because our procedure is unique and has previously been validated with SPM8 ([Takeuchi et al., 2013](#_ENREF_29)). Furthermore, when we use SPM12 and the same parameter sets that were validated in SPM8, apparent misclassifications of tissue types in certain brain areas repeatedly occur during the segmentation processes. The description in this subsection is largely reproduced from our previous study ([Takeuchi et al., 2021](#_ENREF_27)).

In contrast, in the second-level analyses, SPM5 was used because BPM, which uniquely allows voxel-by-voxel correction of multiple image values, is only available in SPM5. Note that similar results were obtained in the non-whole-brain imaging analyses.

**Comparisons of the effects of diagnosis of diabetes on outcome measures in the main analyses, and how the statistical values of effects of BMI on outcome measures change when the covariate of diagnosis of diabetes is excluded.**

To evaluate the effects of a diabetes diagnosis at baseline on the relationship between BMI and cognitive outcomes, we compared the effects of diagnosis of diabetes at baseline in each psychological analysis of main analyses as well as total or mean imaging measures (e.g., total GMV, mean MD in the areas of analyses), the effects of BMI on each measure with and without the covariate of diabetes.

**Evaluation of the effects hyperlipidemia at baseline on outcome measures in supplemental analyses including the covariate of hyperlipidemia at baseline, and how the effects of BMI on outcome measures change with respect from the main analyses**

In this evaluation, we created the composite variable of existence of hyperlipidemia. Existence of hyperlipidemia was defined as self-reported high cholesterol at assessment, as well as diagnoses in HES records (ICD-9 272.0; ICD-10 E78.0) (ICD-9 272/ICD-10 E78) before the assessment, as previously reported ([Inouye et al., 2018](#_ENREF_14)).

We conducted supplemental analyses including this composite variable in addition to all other covariates in each of the main analyses. In imaging analyses, we used the total imaging value (total rGMV, total FA) for independent and dependent imaging measures.

**Evaluation of the impact of excluding subjects with comorbidities on the effects of BMI on outcome measures**

Next, we investigated how the effects of BMI on each measure change when we only remove subjects with cancer and other serious medical condition/disability, as well as when we remove subjects with major complications of obesity, cancers, and other serious medical conditions/disabilities.

In these analyses, as in the analyses of diabetes and hyperlipidemia, we used total or mean imaging values for imaging independent and dependent variables for imaging analyses.

In the former analyses, we only removed subjects with a medical diagnosis of cancer (UK Biobank data field ID:2453) and medical diagnosis of other serious medical condition/disability (UK Biobank data field ID:2473). In addition, we re-ran the main analyses with same covariates as those of the main analyses. In the latter analyses, we removed subjects who had hyperlipidemia (defined in the abovementioned analyses of hyperlipidemia), diabetes (defined in the analyses of diabetes), doctor diagnosis of heart attack, stroke, high blood pressure, angina (UK Biobank data field ID:6150), doctor diagnosis of blood clot in the leg, blood clot in the lung, emphysema/chronic bronchitis (UK Biobank data field ID:6152), doctor diagnosis of cancer (UK Biobank data field ID:2453), doctor diagnosis of other serious medical condition/disability (UK Biobank data field ID:2473). Finally, we re-ran the main analyses with the same covariates except that diagnosis of diabetes was removed from the covariates.

**Supplemental analyses of effects of interaction between BMI and age**

To evaluate the possible effects of an interaction between BMI and age on outcome variables, we conducted supplemental analyses on the interaction between BMI and age group (60 > x, or x ≥ 60, years old). In the prospective analysis of dementia, we conducted an additional cox hazard regression analysis where the additional covariates of age group (60 > x, or x ≥ 60, years old), as well as the interaction term of age group * BMI was added in addition to all the variables used in the main analysis of the effect of the continuous variable of BMI on incident dementia. In the psychological analyses, we conducted additional ANCOVAs, where the fixed factor of age group (60 > x, or x ≥ 60, years old), as well as the interaction term of age group * BMI was added in addition to all the variables used in the main multiple regression analyses of effects of BMI on psychological variables. Furthermore, in imaging analyses, we conducted additional ANCOVAs similar to those of psychological analyses, but in case of imaging analyses all dependent and independent variables correspond to the total (GMV or WMV) or mean (diffusion measures) values of imaging measures as are the cases of the abovementioned evaluation of diabetes effects.

**Supplemental Results**

**Comparisons of the effects of diagnosis of diabetes on outcome measures in the main analyses, and how the statistical values of effects of BMI on outcome measures change when the covariate of diagnosis of diabetes is excluded.**

In analyses of cognitive measures, diagnosis of diabetes was significantly associated with the greater reaction time increase, greater depressive tendency increase, but not with changes of fluid intelligence. Analyses removing the covariate of diagnosis of diabetes at baseline, remained results of fluid intelligence insignificant and remained results of depressive tendency scores strongly significant, but made the results of the positive associations of BMI and reaction time change substantially stronger (p value changed from 0.042, uncorrected, to p = 0.009, uncorrected)

In the main analysis of dementia, diagnosis of diabetes at baseline was significantly associated with the increase of risk of subsequent dementia onset. Removing this covariate made the significant associations of BMI and risk of subsequent dementia onset in main analyses using the categorical variable of BMI, weaker, but remained it significant (p = 9.9*10^-5^ became 0.001, uncorrected).

In analyses of total and mean variables of neuroimaging measures, diagnosis of diabetes at baseline was not significantly associated with any measures, and removing this covariate affected did not significantly alter the results of effects of BMI.

For statistical values, see Supplemental Table 8.

**Supplemental analyses of effects of interaction between BMI and age.**

There were no significant interaction effects in any interaction analyses of effects of the interaction between BMI and age group (all, p > 0.1).

**Supplemental Discussion**

**The possible micro-level mechanisms behind neuroimaging findings**

Observed neuroimaging changes associated with higher BMI mostly indicate relative increase (less decrease) of various tissue components. Potential correlates of rGMV include the number and size of neurons and glia, the level of synaptic bulk, and neurites ([May and Gaser, 2006](#_ENREF_18)). Potential rWMV correlates may include number of glial cells (forming a major portion of white matter) and the number of axon collateral spines, which may selectively increase rWMV while leaving FA relatively unchanged ([Takeuchi et al., 2011](#_ENREF_28)). Areas of increased FA may reflect relative increases of myelin, and axonal membrane thickness and diameter ([Beaulieu, 2002](#_ENREF_4)). NODDI allows for more selective evaluation of tissue components and relative increase of ICVF is supposed to reflect increase of neurite density (axon, dendrites) ([Deligianni et al., 2016](#_ENREF_11)). ISOVF represents the relative amount of extracellular free water diffusion, interstitial fluids, and CSF ([Sepehrband et al., 2015](#_ENREF_23)). As our analyses removed CSF areas thoroughly and regressed out effects of regional CSF density at each voxel, present results may reflect relative increase of interstitial fluids following higher BMI due to increase of tissue components. The relative decline of fluid components and increase of tissue components might lead to uniform decreases of MD/AD/RD, which suggests unidirectional decline of water diffusion in the areas. However, maladaptive changes such as vessel or capillary occlusions might contribute to decrease of isotropic water diffusions and lead to decrease of ISOVF and MD/AD/RD.

**The possibe reasons why lower BMI in late life seems to precede the accelerated neural changes observed in aging and dementia**

The reasons why lower BMI in late life seems to precede the accelerated neural changes observed in aging and dementia, are not clear. Various factors might explain the association between a higher BMI and lower risk of late life dementia ([Qizilbash et al., 2015](#_ENREF_21)). These factors mostly involve nutrient factors and include higher BMI leads to higher late-life blood pressure, high late-life cholesterol, higher leptin, age-related regulatory changes in carbohydrate, lipid or protein metabolisms, higher vitamin E, and vitamin D. These factors are supposed to protect against dementia through various mechanisms, including more nutrients for neural mechanisms, and antioxidant effects. Interestingly, an animal imaging study suggested that IL-6, which is partly produced by adipose tissues may counteract neurodegenerative effects of amyloid beta ([Colon-Perez et al., 2019](#_ENREF_8)). Through some of these mechanisms, a higher BMI may precede the opposite neural mechanisms and neuroimaging variables to those observed in aging and dementia. Another possibility is that the pre-clinical condition of Alzheimer’s disease may involve weight loss and such a state may show accelerated neural atrophy or aging. Non-demented non-obese adults show lower amyloid beta and tau levels at baseline and lower risk of subsequent development of dementia ([Sun et al., 2020](#_ENREF_25)).

**The possible reasons why the associations between greater BMI and greater hippocampal volume decline as well as increase in depressive tendency are seen**

Higher BMI may lead to body dissatisfaction, which in turn leads to lower self-esteem, which may increase depressive moods and thus increase risk of depressive tendencies ([Choi and Choi, 2016](#_ENREF_7)). Alternatively, adipose tissue produces pro-inflammatory molecules, and obese individuals show elevated levels of these pro-inflammatory molecules such as IL-1, IL-6 and C-reactive proteins. And these cytokines are linked to neurodegeneration in the hippocampus ([for summary, see Cherbuin et al., 2015](#_ENREF_6)). In addition, some of these cytokines are suggested to be involved in generation and maintenance of depression ([Jeon and Kim, 2016](#_ENREF_15)). Thus, through these mechanisms, higher BMI may be associated with hippocampal volume reduction and increase of depressive tendency. However, these are speculations and future studies using experimental methods have to ascertain these issues.

**References**

Alfaro-Almagro, F., Jenkinson, M., Bangerter, N.K., Andersson, J.L., Griffanti, L., Douaud, G., Sotiropoulos, S.N., Jbabdi, S., Hernandez-Fernandez, M., and Vallee, E. (2018). Image processing and Quality Control for the first 10,000 brain imaging datasets from UK Biobank. *Neuroimage* 166**,** 400-424.

Ashburner, J., and Friston, K.J. (2000). Voxel-based morphometry-the methods. *Neuroimage* 11**,** 805-821.

Batty, G.D., Mcintosh, A.M., Russ, T.C., Deary, I.J., and Gale, C.R. (2016). Psychological distress, neuroticism, and cause-specific mortality: early prospective evidence from UK Biobank. *J Epidemiol Community Health* 70**,** 1136-1139.

Beaulieu, C. (2002). The basis of anisotropic water diffusion in the nervous system–a technical review. *NMR in Biomedicine* 15**,** 435-455.

Celis-Morales, C.A., Lyall, D.M., Steell, L., Gray, S.R., Iliodromiti, S., Anderson, J., Mackay, D.F., Welsh, P., Yates, T., and Pell, J.P. (2018). Associations of discretionary screen time with mortality, cardiovascular disease and cancer are attenuated by strength, fitness and physical activity: findings from the UK Biobank study. *BMC medicine* 16**,** 77.

Cherbuin, N., Sargent-Cox, K., Fraser, M., Sachdev, P., and Anstey, K. (2015). Being overweight is associated with hippocampal atrophy: the PATH Through Life Study. *International Journal of Obesity* 39**,** 1509-1514.

Choi, E., and Choi, I. (2016). The associations between body dissatisfaction, body figure, self-esteem, and depressed mood in adolescents in the United States and Korea: A moderated mediation analysis. *Journal of Adolescence* 53**,** 249-259.

Colon-Perez, L.M., Ibanez, K.R., Suarez, M., Torroella, K., Acuna, K., Ofori, E., Levites, Y., Vaillancourt, D.E., Golde, T.E., and Chakrabarty, P. (2019). Neurite orientation dispersion and density imaging reveals white matter and hippocampal microstructure changes produced by Interleukin-6 in the TgCRND8 mouse model of amyloidosis. *Neuroimage* 202**,** 116138.

Cullen, B., Newby, D., Lee, D., Lyall, D.M., Nevado-Holgado, A.J., Evans, J.J., Pell, J.P., Lovestone, S., and Cavanagh, J. (2018). Cross-sectional and longitudinal analyses of outdoor air pollution exposure and cognitive function in UK Biobank. *Scientific reports* 8**,** 1-14.

Daducci, A., Canales-Rodríguez, E.J., Zhang, H., Dyrby, T.B., Alexander, D.C., and Thiran, J.-P. (2015). Accelerated microstructure imaging via convex optimization (AMICO) from diffusion MRI data. *NeuroImage* 105**,** 32-44.

Deligianni, F., Carmichael, D.W., Zhang, G.H., Clark, C.A., and Clayden, J.D. (2016). NODDI and tensor-based microstructural indices as predictors of functional connectivity. *PLoS One* 11**,** e0153404.

Hamer, M., Yates, T., and Demakakos, P. (2017). Television viewing and risk of mortality: exploring the biological plausibility. *Atherosclerosis* 263**,** 151-155.

Howe, L.J., Lawson, D.J., Davies, N.M., Pourcain, B.S., Lewis, S.J., Smith, G.D., and Hemani, G. (2019). Genetic evidence for assortative mating on alcohol consumption in the UK Biobank. *Nature communications* 10**,** 1-10.

Inouye, M., Abraham, G., Nelson, C.P., Wood, A.M., Sweeting, M.J., Dudbridge, F., Lai, F.Y., Kaptoge, S., Brozynska, M., and Wang, T. (2018). Genomic risk prediction of coronary artery disease in 480,000 adults: implications for primary prevention. *Journal of the American College of Cardiology* 72**,** 1883-1893.

Jeon, S.W., and Kim, Y.K. (2016). Neuroinflammation and cytokine abnormality in major depression: cause or consequence in that illness? *World journal of psychiatry* 6**,** 283-293.

Khubchandani, J., Brey, R., Kotecki, J., Kleinfelder, J., and Anderson, J. (2016). The psychometric properties of PHQ-4 depression and anxiety screening scale among college students. *Archives of psychiatric nursing* 30**,** 457-462.

Lourida, I., Hannon, E., Littlejohns, T.J., Langa, K.M., Hyppönen, E., Kuźma, E., and Llewellyn, D.J. (2019). Association of lifestyle and genetic risk with incidence of dementia. *Jama* 322**,** 430-437.

May, A., and Gaser, C. (2006). Magnetic resonance-based morphometry: a window into structural plasticity of the brain. *Current Opinion in Neurology* 19**,** 407-411.

Miller, K.L., Alfaro-Almagro, F., Bangerter, N.K., Thomas, D.L., Yacoub, E., Xu, J., Bartsch, A.J., Jbabdi, S., Sotiropoulos, S.N., and Andersson, J.L. (2016). Multimodal population brain imaging in the UK Biobank prospective epidemiological study. *Nature neuroscience* 19**,** 1523-1536.

Okbay, A., Beauchamp, J.P., Fontana, M.A., Lee, J.J., Pers, T.H., Rietveld, C.A., Turley, P., Chen, G.B., Emilsson, V., Meddens, S.F., Oskarsson, S., Pickrell, J.K., Thom, K., Timshel, P., De Vlaming, R., Abdellaoui, A., Ahluwalia, T.S., Bacelis, J., Baumbach, C., Bjornsdottir, G., Brandsma, J.H., Pina Concas, M., Derringer, J., Furlotte, N.A., Galesloot, T.E., Girotto, G., Gupta, R., Hall, L.M., Harris, S.E., Hofer, E., Horikoshi, M., Huffman, J.E., Kaasik, K., Kalafati, I.P., Karlsson, R., Kong, A., Lahti, J., Van Der Lee, S.J., Deleeuw, C., Lind, P.A., Lindgren, K.O., Liu, T., Mangino, M., Marten, J., Mihailov, E., Miller, M.B., Van Der Most, P.J., Oldmeadow, C., Payton, A., Pervjakova, N., Peyrot, W.J., Qian, Y., Raitakari, O., Rueedi, R., Salvi, E., Schmidt, B., Schraut, K.E., Shi, J., Smith, A.V., Poot, R.A., St Pourcain, B., Teumer, A., Thorleifsson, G., Verweij, N., Vuckovic, D., Wellmann, J., Westra, H.J., Yang, J., Zhao, W., Zhu, Z., Alizadeh, B.Z., Amin, N., Bakshi, A., Baumeister, S.E., Biino, G., Bonnelykke, K., Boyle, P.A., Campbell, H., Cappuccio, F.P., Davies, G., De Neve, J.E., Deloukas, P., Demuth, I., Ding, J., Eibich, P., Eisele, L., Eklund, N., Evans, D.M., Faul, J.D., Feitosa, M.F., Forstner, A.J., Gandin, I., Gunnarsson, B., Halldorsson, B.V., Harris, T.B., Heath, A.C., Hocking, L.J., Holliday, E.G., Homuth, G., Horan, M.A., et al. (2016). Genome-wide association study identifies 74 loci associated with educational attainment. *Nature* 533**,** 539-542.

Qizilbash, N., Gregson, J., Johnson, M.E., Pearce, N., Douglas, I., Wing, K., Evans, S.J., and Pocock, S.J. (2015). BMI and risk of dementia in two million people over two decades: a retrospective cohort study. *The lancet Diabetes & endocrinology* 3**,** 431-436.

Sarkar, S.N., Huang, R.-Q., Logan, S.M., Yi, K.D., Dillon, G.H., and Simpkins, J.W. (2008). Estrogens directly potentiate neuronal L-type Ca2+ channels. *Proceedings of the National Academy of Sciences* 105**,** 15148-15153.

Sepehrband, F., Clark, K.A., Ullmann, J.F., Kurniawan, N.D., Leanage, G., Reutens, D.C., and Yang, Z. (2015). Brain tissue compartment density estimated using diffusion‐weighted MRI yields tissue parameters consistent with histology. *Human brain mapping* 36**,** 3687-3702.

Shen, X., Cox, S.R., Adams, M.J., Howard, D.M., Lawrie, S.M., Ritchie, S.J., Bastin, M.E., Deary, I.J., Mcintosh, A.M., and Whalley, H.C. (2018). Resting-state connectivity and its association with cognitive performance, educational attainment, and household income in the UK Biobank. *Biological Psychiatry: Cognitive Neuroscience and Neuroimaging* 3**,** 878-886.

Sun, Z., Wang, Z.-T., Sun, F.-R., Shen, X.-N., Xu, W., Ma, Y.-H., Dong, Q., Tan, L., Yu, J.-T., and Initiative, A.S.D.N. (2020). Late-life obesity is a protective factor for prodromal Alzheimer’s disease: a longitudinal study. *Aging (Albany NY)* 12**,** 2005.

Takeuchi, H., Taki, Y., Hashizume, H., Asano, K., Asano, M., Sassa, Y., Yokota, S., Kotozaki, Y., Nouchi, R., and Kawashima, R. (2016). Impact of videogame play on the brain’s microstructural properties: Cross-sectional and longitudinal analyses. *Molecular Psychiatry* 21**,** 1781-1789.

Takeuchi, H., Taki, Y., Nouchi, R., Yokoyama, R., Kotozaki, Y., Nakagawa, S., Sekiguchi, A., Iizuka, K., Hanawa, S., and Araki, T. (2021). Lead exposure is associated with functional and microstructural changes in the healthy human brain. *Communications Biology* 4**,** 1-14.

Takeuchi, H., Taki, Y., Sassa, Y., Hashizume, H., Sekiguchi, A., Fukushima, A., and Kawashima, R. (2011). Verbal working memory performance correlates with regional white matter structures in the fronto-parietal regions. *Neuropsychologia* 49**,** 3466-3473

Takeuchi, H., Taki, Y., Thyreau, B., Sassa, Y., Hashizume, H., Sekiguchi, A., Nagase, T., Nouchi, R., Fukushima, A., and Kawashima, R. (2013). White matter structures associated with empathizing and systemizing in young adults. *Neuroimage* 77**,** 222-236.

Townsend, P. (1987). Deprivation. *Journal of social policy* 16**,** 125-146.

**Supplemental Table 1.** Baseline characteristics of UK Biobank participants included in the present project (n = 502,505)

| Item | No. (%) | Mean (SD) | Range |
| --- | --- | --- | --- |
| Sex |  |  |  |
| Female | 273,382 (54.4) |  |  |
| Male | 229,122 (45.6) |  |  |
| Missing | 1 |  |  |
| Age, years |  | 56.5 (8.0) | 37-73 |
| Missing | 1 (0.0) |  |  |
| BMI |  | 27.4(4.8) | 12-75 |
| Underweight (18.5≧x) | 2,626 (0.5) |  |  |
| Normal (25≧x>18.5) | 162,523 (32.3) |  |  |
| Overweight (30≧x>25) | 212,097 (42.2) |  |  |
| Obesity (30>x) | 122,153 (24.3) |  |  |
| Missing | 3,107 (0.6) |  |  |
| Average total household income before tax |  |  |  |
| Less than £18,000 | 97,198 (19.3) |  |  |
| £18,000 to £30,999 | 108,177 (21.5) |  |  |
| £31,000 to £5,1999 | 110,772 (22.0) |  |  |
| £52,000 to £100,000 | 86,266 (17.2) |  |  |
| Greater than £100,000 | 22,929 (4.6) |  |  |
| Missing | 77,164 (15.4) |  |  |
| Townsend index of material deprivation |  | -1.3 (3.1) | -6-11 |
| Missing | 624(0.1) |  |  |
| Employment status |  |  |  |
| In paid employment or self-employed | 287,149(57.1) |  |  |
| Not in paid employment or self-employed | 212,404(42.3) |  |  |
| Missing | 2,952(0.6) |  |  |
| Highest education qualification (years) |  | 13.95(5.1) | 7-20 |
| Fluid intelligence |  | 6.0(2.2) | 0-13 |
| Missing | 339,748(67.2) |  |  |

**Supplemental Table 2.** Baseline characteristics of UK Biobank participants included in the imaging analyses (n = 1,253)

| Item | No. (%) | Mean (SD) | Range |
| --- | --- | --- | --- |
| Sex |  |  |  |
| Female | 621 (49.6) |  |  |
| Male | 632 (50.4) |  |  |
| Age, years |  | 62.1 (7.1) | 46-79 |
| BMI |  | 26.2(4.2) | 13-51 |
| Underweight (18.5≧x) | 13 (1.0) |  |  |
| Normal (25≧x>18.5) | 512(40.9) |  |  |
| Overweight (30≧x>25) | 517(41.3) |  |  |
| Obesity (30>x) | 211(16.8) |  |  |
| Average total household income before tax |  |  |  |
| Less than £18,000 | 152 (12.1) |  |  |
| £18,000 to £30,999 | 336 (26.8) |  |  |
| £31,000 to £5,1999 | 391 (31.2) |  |  |
| £52,000 to £100,000 | 288 (23.0) |  |  |
| Greater than £100,000 | 86 (6.9) |  |  |
| Townsend index of material deprivation |  | -2.3 (2.6) | -6-11 |
| Employment status |  |  |  |
| In paid employment or self-employed | 530(42.3) |  |  |
| Not in paid employment or self-employed | 723(57.7) |  |  |
| Highest education qualification (years) |  | 13.95(5.1) | 7-20 |

**Supplemental Table** **3**. Brain regions that exhibited significant correlations between BMI and changes in rGMV in longitudinal analyses.

| Included gray matter areas^1^ (number of significant voxels in left and right side of each anatomical area) | x  y  z | T score | Corrected *p* value (FDR) | Cluster size (mm^3^, corrected cluster level p value)^2^ | r^3^  (p)  BMI*  Img  (change) | r^4^  (p)  BMI*  Img  (pre) | r^5^  (p)  Age*  Img  (pre) |
| --- | --- | --- | --- | --- | --- | --- | --- |
| Positive correlation between BMI and rGMV change |  |  |  |  |  |  |  |
| Cerebellum (L:38)/ | -30  -57  -42 | 4.98 | 0.007 | 1296, 0.008 | 0.046  0.104 | 0.175  4.9*10^-10^ | -0.178  2.0*10^-10^ |
| Inferior temporal gyrus (L:48)/Middle temporal gyrus (L:97)/ | -48  -12  -21 | 4.75 | 0.007 | 3780, <0.001 | 0.122  1.4*10^-5^ | 0.057  0.045 | -0.264  2.2*10^-21^ |
| Angular gyrus (R:16)/Middle occipital lobe (R:14)/Superior occipital lobe (R:1)/ | 39  -78  39 | 4.38 | 0.010 | 756, 0.048 | 0.097  0.001 | 0.139  7.9*10^-7^ | -0.126  0.7*10^-5^ |
| Negative correlation between BMI and rGMV change |  |  |  |  |  |  |  |
| Amygdala (R:38)/Fusiform gyrus (R:12)/Hippocampus (R:2)/Parahippocampal gyrus (R:35)/Temporal pole (R:2)/ | 27  0  -18 | 4.49 | 0.029 | 3591, <0.001 | -0.091  0.001 | -0.028  0.314 | -0.360  1.1*10^-39^ |
| Amygdala (L:6)/Fusiform gyrus (L:2)/Hippocampus (L:28)/ | -30  -9  -21 | 4.47 | 0.029 | 1053, 0.017 | -0.092  0.001 | -0.010  0.718 | -0.286  4.3*10^-25^ |

^1^Labelings of the anatomical regions of gray matter were mostly based on the WFU PickAtlas Tool (<http://www.fmri.wfubmc.edu/cms/software#PickAtlas/>) and on the PickAtlas automated anatomical labeling atlas option. The automated anatomical labeling had subdivided classifications for the cerebellum, but for ensuring simplicity, we combined these subdivided areas and created a mask for the cerebellum and used it here.

^2^Only the clusters that surpassed the extent threshold with the voxel level cluster determining the threshold (*P* < 0.05, corrected for the false discovery rate) were noted.

^3^Simple correlation coefficients of correlation between pre to post-scan changes of mean imaging values of clusters and BMI at pre-scan.

^4^Simple correlation coefficients of correlation between mean imaging values of clusters at pre-scan and BMI at pre-scan.

^5^Simple correlation coefficients of correlation between mean imaging values of clusters at pre-scan and age at pre-scan.

**Supplemental Table** **4**. Brain regions that exhibited significant positive correlations between BMI and changes in

rWMV in longitudinal analyses.

| Included large bundles (number of significant voxels in left and right side of each anatomical area)^1^ | x  y  z | T score | Corrected *p* value (FDR) | Cluster size (mm^3^, corrected cluster level p value)^2^ | r^3^  (p)  BMI*  Img  (change) | r^4^  (p)  BMI*  Img  (pre) | r^5^  (p)  Age*  Img  (pre) |
| --- | --- | --- | --- | --- | --- | --- | --- |
| Middle cerebellar peduncle (312)/Inferior cerebellar peduncle (L:7, R:2)/Superior cerebellar peduncle (L:3, R:2)/ | 30  -60  -51 | 8.20 | <0.001 | 54945, <0.001 | -0.001  0.964 | 0.252  1.2*10^-19^ | 0.032  0.259 |
| Retrolenticular part of internal capsule (L:22)/Posterior thalamic radiation (L:23)/Sagittal stratum (L:66)/External capsule (L:16)/Cingulum (L:25)/Heschl gyrus (L:25)/Stria terminalis (L:10)/Superior longitudinal fasciculus (L:37)/Inferior fronto-occipital fasciculus (L:7)/ | -54  -24  -18 | 6.41 | <0.001 | 71766, <0.001 | 0.102  3.1*10^-4^ | 0.107  1.5*10^-4^ | -0.185  4.1*10^-11^ |
| Genu of corpus callosum (17)/Anterior limb of internal capsule (R:28)/Anterior corona radiata (R:40)/Sagittal stratum (R:24)/External capsule (R:11)/Cingulum (R:13)/Heschl gyrus (R:13)/Stria terminalis (R:13)/Inferior fronto-occipital fasciculus (R:11)/Uncinate fasciculus (R:13)/ | 27  -18  -30 | 5.22 | <0.001 | 36342, <0.001 | 0.106  1.8*10^-4^ | 0.144  2.8*10^-7^ | -0.160  1.2*10^-8^ |

^1^The anatomical labels and significant clusters of major white matter fibers were determined using the ICBM DTI-81 Atlas (<http://www.loni.ucla.edu/>). The automated anatomical labeling had subdivided classifications for the cerebellum, but for ensuring simplicity, we combined these subdivided areas and created a mask for the cerebellum and used it here.

^2^Only the clusters that surpassed the extent threshold with the voxel level cluster determining the threshold (*P* < 0.05, corrected for the false discovery rate) were noted.

^3^Simple correlation coefficients of correlation between pre to post-scan changes of mean imaging values of clusters and BMI at pre-scan.

^4^Simple correlation coefficients of correlation between mean imaging values of clusters at pre-scan and BMI at pre-scan.

^5^Simple correlation coefficients of correlation between mean imaging values of clusters at pre-scan and age at pre-scan.

**Supplemental Table** **5**. Brain regions that exhibited significant positive correlations between BMI and changes in FA in longitudinal analyses.

| Included large bundles (number of significant voxels in left and right side of each anatomical area)^1^ | x  y  z | T score | Corrected *p* value (FDR) | Cluster size (mm^3^, corrected cluster level p value)^2^ | r^3^  (p)  BMI*  Img  (change) | r^4^  (p)  BMI*  Img  (pre) | r^5^  (p)  Age*  Img  (pre) |
| --- | --- | --- | --- | --- | --- | --- | --- |
| Middle cerebellar peduncle (3)/Pontine crossing tract (10)/Medial lemniscus (L:13, R:32)/Superior cerebellar peduncle (R:3)/ | 6  -38  -30 | 5.31 | 0.001 | 536, 0.003 | 0.099  4.7*10^-4^ | 0.093  0.001 | 0.077  0.001 |
| Posterior limb of internal capsule (L:61)/Retrolenticular part of internal capsule (L:18)/ | -26  -18  -2 | 4.90 | 0.001 | 912, <0.001 | 0.160  1.6*10^-8^ | -0.067  0.018 | -0.195  4.6*10^-12^ |
| Superior corona radiata (L:4)/Posterior corona radiata (L:71)/ | -20  -34  36 | 4.28 | 0.006 | 1176, <0.001 | 0.133  0.2*10^-5^ | -0.114  5.8*10^-5^ | -0.181  1.2*10^-10^ |
| Superior corona radiata (L:3)/Superior longitudinal fasciculus (L:1)/ | -26  -16  48 | 4.27 | 0.006 | 280, 0.005 | 0.115  5.1*10^-5^ | -0.062  0.025 | -0.072  0.012 |
| None | 50  -20  -18 | 3.96 | 0.010 | 344, 0.024 | 0.120  2.2*10^-5^ | 0.011  0.705 | -0.092  0.001 |

^1^The anatomical labels and significant clusters of major white matter fibers were determined using the ICBM DTI-81 Atlas (<http://www.loni.ucla.edu/>). The automated anatomical labeling had subdivided classifications for the cerebellum, but for ensuring simplicity, we combined these subdivided areas and created a mask for the cerebellum and used it here.

^2^Only the clusters that surpassed the extent threshold with the voxel level cluster determining the threshold (*P* < 0.05, corrected for the false discovery rate) were noted.

^3^Simple correlation coefficients of correlation between pre to post-scan changes of mean imaging values of clusters and BMI at pre-scan.

^4^Simple correlation coefficients of correlation between mean imaging values of clusters at pre-scan and BMI at pre-scan.

^5^Simple correlation coefficients of correlation between mean imaging values of clusters at pre-scan and age at pre-scan.

**Supplemental Table** **6**. Brain regions that exhibited significant negative correlations between BMI and changes in MD/AD/RD in longitudinal analyses.

| Included gray matter areas^1^ (number of significant voxels in left and right side of each anatomical area) | Included large bundles^2^ (number of significant voxels in left and right side of each anatomical area) | x  y  z | T | Corrected  *p* value (FDR) | Cluster  size  (mm^3^,  corrected  cluster  level  p value)^3^ | r^4^  (p)  BMI*  Img  (change) | r^5^  (p)  BMI*  Img  (pre) | r^6^  (p)  Age*  Img  (pre) |
| --- | --- | --- | --- | --- | --- | --- | --- | --- |
| Negative correlation between BMI and change in MD |  |  |  |  |  |  |  |  |
| Amygdala (L:35, R:75)/Angular gyrus (L:286, R:303)/Calcarine Cortex (L:194, R:446)/Caudate (L:34, R:54)/Anterior cingulum (R:9)/Middle cingulum (L:494, R:759)/Posterior cingulum (L:290, R:216)/Cuneus (L:215, R:359)/Inferior frontal operculum (R:202)/Inferior frontal orbital area (R:20)/Inferior frontal triangular (R:131)/Middle frontal medial area (R:50)/Middle frontal orbital area (R:26)/Superior frontal orbital area (R:94)/Fusiform gyrus (L:751, R:1130)/Heschl gyrus (L:4, R:27)/Hippocampus (L:153, R:260)/Insula (L:53, R:207)/Lingual gyrus (L:679, R:825)/Inferior occipital lobe (L:513, R:369)/Middle occipital lobe (L:1444, R:737)/Superior occipital lobe (L:329, R:452)/Pallidum (L:70, R:81)/Parahippocampal gyrus (L:269, R:435)/Inferior parietal lobule (L:328, R:45)/Superior parietal lobule (L:158, R:19)/Postcentral gyrus (L:292, R:322)/Precentral gyrus (L:7, R:24)/Precuneus (L:780, R:978)/Putamen (L:139, R:136)/Rectus gyrus (R:117)/Rolandic operculum (L:109, R:204)/Supplemental motor area (R:1)/Supramarginal gyrus (L:317, R:352)/Inferior temporal gyrus (L:472, R:1349)/Middle temporal gyrus (L:1228, R:1619)/Temporal pole (R:7)/Superior temporal gyrus (L:475, R:982)/Thalamus (L:460, R:628)/Thalamus (L:3694, R:4167)/ | Middle cerebellar peduncle (1348)/Pontine crossing tract (141)/Genu of corpus callosum (3)/Body of corpus callosum (171)/Splenium of corpus callosum (696)/Corticospinal tract (L:85, R:116)/Medial lemniscus (L:82, R:78)/Inferior cerebellar peduncle (L:87, R:99)/Superior cerebellar peduncle (L:82, R:66)/Cerebral peduncle (L:173, R:241)/Anterior limb of internal capsule (L:89, R:133)/Posterior limb of internal capsule (L:383, R:302)/Retrolenticular part of internal capsule (L:189, R:176)/Anterior corona radiata (R:163)/Superior corona radiata (L:210, R:217)/Posterior corona radiata (L:304, R:291)/Posterior thalamic radiation (L:272, R:174)/Sagittal stratum (L:148, R:187)/External capsule (L:90, R:74)/Cingulum (L:230, R:287)/Heschl gyrus (L:118, R:76)/Stria terminalis (L:71, R:72)/Superior longitudinal fasciculus (L:396, R:669)/Superior fronto-occipital fasciculus (L:4)/Inferior fronto-occipital fasciculus (L:7, R:36)/Uncinate fasciculus (R:14)/Tapatum (L:6, R:5)/ | 6  -38  -28 | 7.55 | <0.001 | 401224  <0.001 | -0.121  2.0*10^-5^ | -0.013  0.650 | 0.430  6.7*10^-57^ |
| Negative correlation between BMI and change in AD |  |  |  |  |  |  |  |  |
| Amygdala (L:45, R:83)/Angular gyrus (L:272, R:271)/Calcarine Cortex (L:194, R:451)/Caudate (L:30, R:61)/Anterior cingulum (L:23, R:5)/Middle cingulum (L:590, R:834)/Posterior cingulum (L:301, R:244)/Cuneus (L:242, R:366)/Inferior frontal operculum (L:52, R:193)/Inferior frontal orbital area (R:68)/Inferior frontal triangular (L:4, R:73)/Middle frontal medial area (R:99)/Middle frontal orbital area (R:44)/Superior frontal orbital area (R:162)/Fusiform gyrus (L:777, R:1087)/Heschl gyrus (L:16, R:18)/Hippocampus (L:148, R:250)/Insula (L:96, R:207)/Lingual gyrus (L:678, R:826)/Inferior occipital lobe (L:483, R:369)/Middle occipital lobe (L:1478, R:740)/Superior occipital lobe (L:391, R:473)/Pallidum (L:88, R:102)/Paracentral lobule (L:11, R:2)/Parahippocampal gyrus (L:269, R:417)/Inferior parietal lobule (L:304, R:43)/Superior parietal lobule (L:165, R:17)/Postcentral gyrus (L:359, R:425)/Precentral gyrus (L:5, R:35)/Precuneus (L:825, R:985)/Putamen (L:147, R:178)/Rectus gyrus (R:130)/Rolandic operculum (L:179, R:258)/Supramarginal gyrus (L:309, R:274)/Inferior temporal gyrus (L:501, R:1300)/Middle temporal gyrus (L:1505, R:1675)/Temporal pole (L:6, R:44)/Superior temporal gyrus (L:597, R:1004)/Thalamus (L:571, R:618)/Thalamus (L:3227, R:3720)/ | Middle cerebellar peduncle (1331)/Pontine crossing tract (144)/Genu of corpus callosum (4)/Body of corpus callosum (310)/Splenium of corpus callosum (838)/Corticospinal tract (L:72, R:105)/Medial lemniscus (L:81, R:78)/Inferior cerebellar peduncle (L:75, R:77)/Superior cerebellar peduncle (L:82, R:66)/Cerebral peduncle (L:233, R:242)/Anterior limb of internal capsule (L:84, R:146)/Posterior limb of internal capsule (L:366, R:274)/Retrolenticular part of internal capsule (L:276, R:162)/Anterior corona radiata (R:114)/Superior corona radiata (L:97, R:102)/Posterior corona radiata (L:284, R:228)/Posterior thalamic radiation (L:279, R:167)/Sagittal stratum (L:216, R:187)/External capsule (L:87, R:96)/Cingulum (L:274, R:313)/Heschl gyrus (L:115, R:82)/Stria terminalis (L:97, R:76)/Superior longitudinal fasciculus (L:442, R:545)/Inferior fronto-occipital fasciculus (L:11, R:68)/Uncinate fasciculus (L:40, R:39)/Tapatum (L:8, R:8)/ | 56  -34  -10 | 6.94 | <0.001 | 404608 <0.001 | -0.115  4.6*10^-5^ | -0.057  0.044 | 0.341  3.3*10^-35^ |
| Caudate (L:51)/Anterior cingulum (L:90, R:1)/Inferior frontal orbital area (L:299)/Inferior frontal triangular (L:13)/Middle frontal medial area (L:43)/Middle frontal orbital area (L:205)/Superior frontal orbital area (L:108)/Insula (L:10)/Putamen (L:4)/Rectus gyrus (L:132)/ | Genu of corpus callosum (237)/Body of corpus callosum (5)/Anterior corona radiata (L:18)/Inferior fronto-occipital fasciculus (L:2)/ | -4  26  -10 | 4.12 | <0.001 | 10816, 0.001 | -0.084  0.003 | -0.009  0.761 | 0.354  7.4*10^-38^ |
| Negative correlation between BMI and change in RD |  |  |  |  |  |  |  |  |
| Amygdala (L:39, R:41)/Angular gyrus (L:252, R:277)/Calcarine Cortex (L:135, R:350)/Caudate (L:36, R:32)/Middle cingulum (L:407, R:673)/Posterior cingulum (L:252, R:145)/Cuneus (L:116, R:235)/Fusiform gyrus (L:748, R:1141)/Heschl gyrus (L:2, R:21)/Hippocampus (L:115, R:223)/Insula (L:33, R:53)/Lingual gyrus (L:644, R:821)/Inferior occipital lobe (L:504, R:369)/Middle occipital lobe (L:1121, R:668)/Superior occipital lobe (L:156, R:342)/Pallidum (L:39, R:60)/Parahippocampal gyrus (L:256, R:382)/Inferior parietal lobule (L:288, R:40)/Superior parietal lobule (L:119, R:17)/Postcentral gyrus (L:193, R:263)/Precentral gyrus (L:23, R:14)/Precuneus (L:691, R:880)/Putamen (L:131, R:108)/Rolandic operculum (L:21, R:75)/Supplemental motor area (R:2)/Supramarginal gyrus (L:273, R:361)/Inferior temporal gyrus (L:453, R:1359)/Middle temporal gyrus (L:881, R:1524)/Superior temporal gyrus (L:316, R:910)/Thalamus (L:389, R:593)/Thalamus (L:3363, R:4240)/ | Middle cerebellar peduncle (1011)/Pontine crossing tract (142)/Body of corpus callosum (76)/Splenium of corpus callosum (345)/Corticospinal tract (L:71, R:112)/Medial lemniscus (L:82, R:78)/Inferior cerebellar peduncle (L:68, R:94)/Superior cerebellar peduncle (L:40, R:63)/Cerebral peduncle (L:31, R:168)/Anterior limb of internal capsule (L:75, R:105)/Posterior limb of internal capsule (L:394, R:282)/Retrolenticular part of internal capsule (L:96, R:152)/Superior corona radiata (L:201, R:195)/Posterior corona radiata (L:275, R:268)/Posterior thalamic radiation (L:159, R:144)/Sagittal stratum (L:64, R:183)/External capsule (L:79, R:56)/Cingulum (L:178, R:223)/Heschl gyrus (L:97, R:48)/Stria terminalis (L:23, R:42)/Superior longitudinal fasciculus (L:336, R:659)/Superior fronto-occipital fasciculus (L:7)/Inferior fronto-occipital fasciculus (L:7, R:31)/Tapatum (L:5)/ | 6  -38  -28 | 7.54 | <0.001 | 336000, <0.001 | -0.123  1.4*10^-5^ | -0.002  0.954 | 0.455  1.8*10^-64^ |
| Anterior cingulum (R:14)/Inferior frontal operculum (R:182)/Inferior frontal orbital area (R:1)/Inferior frontal triangular (R:141)/Middle frontal medial area (R:54)/Middle frontal orbital area (R:18)/Superior frontal orbital area (R:44)/Insula (R:81)/Rectus gyrus (R:104)/Rolandic operculum (R:4)/ | Genu of corpus callosum (8)/Anterior corona radiata (R:110)/ | 4  26  -14 | 4.30 | <0.001 | 8488, 0.003 | -0.127  0.7*10^-5^ | 0.095  0.001 | 0.495  8.8*10^-78^ |

^1^Labelings of the anatomical regions of gray matter were mostly based on the WFU PickAtlas Tool (<http://www.fmri.wfubmc.edu/cms/software#PickAtlas/>) and on the PickAtlas automated anatomical labeling atlas option. The automated anatomical labeling had subdivided classifications for the cerebellum, but for ensuring simplicity, we combined these subdivided areas and created a mask for the cerebellum and used it here.

^2^The anatomical labels and significant clusters of major white matter fibers were determined using the ICBM DTI-81 Atlas (<http://www.loni.ucla.edu/>).

^3^Only the clusters that surpassed the extent threshold with the voxel level cluster determining the threshold (*P* < 0.05, corrected for the false discovery rate) were noted.

^4^Simple correlation coefficients of correlation between pre to post-scan changes of mean imaging values of clusters and BMI at pre-scan.

^5^Simple correlation coefficients of correlation between mean imaging values of clusters at pre-scan and BMI at pre-scan.

^6^Simple correlation coefficients of correlation between mean imaging values of clusters at pre-scan and age at pre-scan.

**Supplemental Table** **7**. Brain regions that exhibited significant correlations between BMI and changes in ISOVF in longitudinal analyses.

| Included gray matter areas^1^ (number of significant voxels in left and right side of each anatomical area) | Included large bundles^2^ (number of significant voxels in left and right side of each anatomical area) | x  y  z | T | Corrected *p* value (FDR) | Cluster size (mm^3^, corrected cluster level p value)^3^ | r^4^  (p)  BMI*  Img  (change) | r^5^  (p)  BMI*  Img  (pre) | r^6^  (p)  Age*  Img  (pre) |
| --- | --- | --- | --- | --- | --- | --- | --- | --- |
| Positive correlation between BMI and change in ISOVF |  |  |  |  |  |  |  |  |
| Cerebellum (L:420)/ | Middle cerebellar peduncle (2)/ | -22  -52  -54 | 6.33 | <0.001 | 3464 <0.001 | −0.103  2.7*10^−4^ | 0.245  2.2*10^−18^ | 0.100  4.2*10^−4^ |
| Caudate (L:16)/Pallidum (L:56)/Putamen (L:11)/ | Anterior limb of internal capsule (L:13)/ | -12  2  -8 | 4.98 | 0.001 | 1456  <0.001 | 0.083  0.003 | 0.117  3.5*10^−5^ | 0.201  8.8*10^−13^ |
| Cerebellum (R:326)/ | None | 20  -56  -52 | 4.92 | 0.002 | 2608 <0.001 | -0.046  0.102 | 0.187  3.3*10^−11^ | 0.115  4.7*10^−5^ |
| Negative correlation between BMI and change in ISOVF |  |  |  |  |  |  |  |  |
| Thalamus (L:9, R:246)/ | Middle cerebellar peduncle (614)/Pontine crossing tract (74)/Corticospinal tract (L:11, R:33)/Medial lemniscus (L:51, R:66)/Inferior cerebellar peduncle (L:7, R:20)/Superior cerebellar peduncle (L:40, R:46)/Cerebral peduncle (L:9, R:53)/ | 8  -38  -28 | 6.52 | <0.001 | 11696  <0.001 | -0.092  0.001 | −0.144  3.7*10^−7^ | -0.027  0.340 |
| Angular gyrus (R:209)/Calcarine Cortex (R:274)/Middle cingulum (R:445)/Posterior cingulum (L:11, R:117)/Cuneus (R:289)/Fusiform gyrus (R:591)/Hippocampus (R:43)/Insula (R:4)/Lingual gyrus (R:418)/Inferior occipital lobe (R:356)/Middle occipital lobe (R:592)/Superior occipital lobe (R:488)/Parahippocampal gyrus (R:17)/Inferior parietal lobule (R:34)/Superior parietal lobule (R:14)/Postcentral gyrus (R:163)/Precentral gyrus (R:58)/Precuneus (R:662)/Putamen (R:10)/Supramarginal gyrus (R:128)/Inferior temporal gyrus (R:698)/Middle temporal gyrus (R:1038)/Superior temporal gyrus (R:119)/ | Body of corpus callosum (20)/Splenium of corpus callosum (227)/Retrolenticular part of internal capsule (R:135)/Superior corona radiata (R:88)/Posterior corona radiata (R:177)/Posterior thalamic radiation (R:161)/Sagittal stratum (R:169)/External capsule (R:16)/Cingulum (R:149)/Heschl gyrus (R:6)/Stria terminalis (R:11)/Superior longitudinal fasciculus (R:255)/Inferior fronto-occipital fasciculus (R:2)/ | 46  -32  -8 | 6.35 | <0.001 | 88624  <0.001 | −0.140  7.8*10^−7^ | 0.005  0.865 | 0.394  2.1*10^−47^ |
| Angular gyrus (L:216)/Calcarine Cortex (L:90)/Middle cingulum (L:165)/Posterior cingulum (L:1)/Cuneus (L:161)/Fusiform gyrus (L:435)/Lingual gyrus (L:78)/Inferior occipital lobe (L:143)/Middle occipital lobe (L:885)/Superior occipital lobe (L:381)/Inferior parietal lobule (L:215)/Superior parietal lobule (L:114)/Postcentral gyrus (L:1)/Precuneus (L:304)/Supramarginal gyrus (L:111)/Inferior temporal gyrus (L:272)/Middle temporal gyrus (L:555)/Superior temporal gyrus (L:44)/ | Body of corpus callosum (4)/Splenium of corpus callosum (24)/Superior corona radiata (L:1)/Posterior corona radiata (L:132)/Posterior thalamic radiation (L:118)/Sagittal stratum (L:27)/Cingulum (L:17)/Superior longitudinal fasciculus (L:104)/ | -22  -62  34 | 5.08 | <0.001 | 45104  <0.001 | −0.116  4.5*10^−5^ | 0.029  0.313 | 0.408  4.5*10^−51^ |
| Inferior frontal orbital area (L:30)/Inferior frontal triangular (L:8)/Middle frontal medial area (L:2)/Middle frontal orbital area (L:88)/Middle frontal other areas (L:16)/Superior frontal orbital area (L:43)/Superior frontal other areas (L:2)/Rectus gyrus (L:37)/ | Anterior corona radiata (L:41)/ | -20  36  -10 | 3.78 | 0.002 | 3672  <0.001 | -0.101  3.5*10^-4^ | 0.079  0.005 | 0.215  2.2*10^-14^ |

^1^Labelings of the anatomical regions of gray matter were mostly based on the WFU PickAtlas Tool (<http://www.fmri.wfubmc.edu/cms/software#PickAtlas/>) and on the PickAtlas automated anatomical labeling atlas option. The automated anatomical labeling had subdivided classifications for the cerebellum, but for ensuring simplicity, we combined these subdivided areas and created a mask for the cerebellum and used it here.

^2^The anatomical labels and significant clusters of major white matter fibers were determined using the ICBM DTI-81 Atlas (<http://www.loni.ucla.edu/>).

^3^Only the clusters that surpassed the extent threshold with the voxel level cluster determining the threshold (*P* < 0.05, corrected for the false discovery rate) were noted.

^4^Simple correlation coefficients of correlation between pre to post-scan changes of mean imaging values of clusters and BMI at pre-scan.

^5^Simple correlation coefficients of correlation between mean imaging values of clusters at pre-scan and BMI at pre-scan.

^6^Simple correlation coefficients of correlation between mean imaging values of clusters at pre-scan and age at pre-scan

**Supplemental Table** **8**. Brain regions that exhibited significant correlations between BMI and changes in ICVF and OD in longitudinal analyses.

| Included gray matter areas^1^ (number of significant voxels in left and right side of each anatomical area) | Included large bundles^2^ (number of significant voxels in left and right side of each anatomical area) | x  y  z | T | Corrected *p* value (FDR) | Cluster size (mm^3^, corrected cluster level p value)^3^ | r^4^  (p)  BMI*  Img  (change) | r^5^  (p)  BMI*  Img  (pre) | r^6^  (p)  Age*  Img  (pre) |
| --- | --- | --- | --- | --- | --- | --- | --- | --- |
| Positive correlation between BMI and change in ICVF |  |  |  |  |  |  |  |  |
| Amygdala (L:100, R:122)/Angular gyrus (R:10)/Calcarine Cortex (L:88, R:7)/Caudate (L:201, R:365)/Anterior cingulum (L:2, R:175)/Middle cingulum (L:173)/Posterior cingulum (L:72)/Inferior frontal operculum (R:125)/Inferior frontal orbital area (R:130)/Inferior frontal triangular (R:26)/Middle frontal medial area (R:78)/Middle frontal orbital area (R:4)/Middle frontal other areas (R:67)/Superior frontal medial area (R:7)/Superior frontal orbital area (R:15)/Fusiform gyrus (L:663, R:968)/Heschl gyrus (L:44, R:13)/Hippocampus (L:235, R:189)/Insula (L:199, R:196)/Lingual gyrus (L:514, R:498)/Inferior occipital lobe (L:218, R:168)/Middle occipital lobe (L:409, R:103)/Superior occipital lobe (L:8)/Pallidum (L:235, R:247)/Parahippocampal gyrus (L:301, R:665)/Postcentral gyrus (R:22)/Precuneus (L:336, R:4)/Putamen (L:615, R:701)/Rectus gyrus (R:54)/Rolandic operculum (L:12, R:74)/Supramarginal gyrus (L:13, R:113)/Inferior temporal gyrus (L:344, R:1279)/Middle temporal gyrus (L:381, R:970)/Temporal pole (R:89)/Superior temporal gyrus (L:145, R:642)/Thalamus (L:250, R:507)/Thalamus (L:4006, R:4260)/ | Middle cerebellar peduncle (453)/Pontine crossing tract (137)/Genu of corpus callosum (26)/Splenium of corpus callosum (38)/Corticospinal tract (L:31, R:100)/Medial lemniscus (L:80, R:78)/Inferior cerebellar peduncle (L:69, R:93)/Superior cerebellar peduncle (L:51, R:63)/Cerebral peduncle (L:73, R:70)/Anterior limb of internal capsule (L:169, R:216)/Posterior limb of internal capsule (L:176, R:136)/Retrolenticular part of internal capsule (L:142)/Anterior corona radiata (R:126)/Superior corona radiata (L:54, R:30)/Posterior corona radiata (L:93)/Posterior thalamic radiation (L:54)/Sagittal stratum (L:4, R:46)/External capsule (L:198, R:175)/Cingulum (L:92, R:69)/Heschl gyrus (L:90, R:69)/Stria terminalis (L:12, R:6)/Superior longitudinal fasciculus (L:78, R:38)/Superior fronto-occipital fasciculus (R:21)/Inferior fronto-occipital fasciculus (L:25, R:206)/Uncinate fasciculus (R:28)/Tapatum (L:2)/ | 22  -70  -48 | 7.12 | <0.001 | 230992  0.001 | 0.065  0.023 | 0.160  1.5*10^-8^ | -0.157  2.5*10^-8^ |
| Calcarine Cortex (R:53)/Middle cingulum (R:416)/Posterior cingulum (R:43)/Lingual gyrus (R:1)/Precuneus (R:196)/ | Body of corpus callosum (14)/Splenium of corpus callosum (56)/Cingulum (R:76)/Heschl gyrus (R:1)/Tapatum (R:5)/ | 4  -36  42 | 5.04 | <0.001 | 5936  0.004 | 0.114  5.9*10^-5^ | 0.011  0.699 | -0.219  6.6*10^-15^ |
| Positive correlation between BMI and change in OD |  |  |  |  |  |  |  |  |
| Amygdala (L:28, R:49)/Angular gyrus (L:19, R:15)/Calcarine Cortex (L:155, R:366)/Caudate (L:56, R:193)/Middle cingulum (L:104, R:116)/Posterior cingulum (L:168, R:183)/Cuneus (L:218, R:151)/Inferior frontal operculum (L:41)/Inferior frontal orbital area (R:143)/Middle frontal medial area (R:21)/Middle frontal orbital area (R:41)/Superior frontal orbital area (R:152)/Fusiform gyrus (L:211, R:385)/Heschl gyrus (R:1)/Hippocampus (L:89, R:152)/Insula (L:63, R:107)/Lingual gyrus (L:350, R:384)/Inferior occipital lobe (R:95)/Middle occipital lobe (L:347, R:240)/Superior occipital lobe (L:212, R:194)/Pallidum (L:155, R:178)/Parahippocampal gyrus (L:149, R:357)/Inferior parietal lobule (L:28, R:11)/Superior parietal lobule (L:29)/Postcentral gyrus (L:48)/Precentral gyrus (L:1)/Precuneus (L:478, R:427)/Putamen (L:264, R:657)/Rectus gyrus (R:18)/Rolandic operculum (L:30)/Supramarginal gyrus (L:22)/Inferior temporal gyrus (L:250, R:691)/Middle temporal gyrus (L:626, R:661)/Temporal pole (L:3, R:24)/Superior temporal gyrus (L:229, R:612)/Thalamus (L:305, R:480)/Thalamus (L:2924, R:3151)/ | Middle cerebellar peduncle (837)/Pontine crossing tract (68)/Genu of corpus callosum (10)/Body of corpus callosum (116)/Splenium of corpus callosum (633)/Corticospinal tract (R:45)/Medial lemniscus (L:61, R:58)/Inferior cerebellar peduncle (L:57, R:54)/Superior cerebellar peduncle (L:77, R:66)/Cerebral peduncle (L:170, R:236)/Anterior limb of internal capsule (L:142, R:232)/Posterior limb of internal capsule (L:217, R:119)/Retrolenticular part of internal capsule (L:65, R:26)/Anterior corona radiata (R:8)/Posterior corona radiata (L:64, R:65)/Posterior thalamic radiation (L:110, R:42)/Sagittal stratum (L:158, R:99)/External capsule (L:41, R:190)/Cingulum (L:139, R:171)/Heschl gyrus (L:67, R:71)/Stria terminalis (L:98, R:42)/Superior longitudinal fasciculus (L:97, R:10)/Inferior fronto-occipital fasciculus (L:57, R:198)/Uncinate fasciculus (L:29, R:35)/Tapatum (R:9)/ | 6  -16  -10 | 6.61 | <0.001 | 210392 <0.001 | 0.085  0.003 | 0.205  2.8*10^-13^ | 0.209  9.5*10^-14^ |

^1^Labelings of the anatomical regions of gray matter were mostly based on the WFU PickAtlas Tool (<http://www.fmri.wfubmc.edu/cms/software#PickAtlas/>) and on the PickAtlas automated anatomical labeling atlas option. The automated anatomical labeling had subdivided classifications for the cerebellum, but for ensuring simplicity, we combined these subdivided areas and created a mask for the cerebellum and used it here.

^2^The anatomical labels and significant clusters of major white matter fibers were determined using the ICBM DTI-81 Atlas (<http://www.loni.ucla.edu/>).

^3^Only the clusters that surpassed the extent threshold with the voxel level cluster determining the threshold (*P* < 0.05, corrected for the false discovery rate) were noted.

^4^Simple correlation coefficients of correlation between pre to post-scan changes of mean imaging values of clusters and BMI at pre-scan.

^5^Simple correlation coefficients of correlation between mean imaging values of clusters at pre-scan and BMI at pre-scan.

^6^Simple correlation coefficients of correlation between mean imaging values of clusters at pre-scan and age at pre-scan

**Supplemental Table 9.** Characteristics of diabetes according to obesity level in the UK Biobank cohort in the 1^st^ assessment visit (baseline for the cognitive analyses) and the 3^rd^ assessment visit (the baseline for the imaging analyses, for those who attended both of third and fourth assessment).

| 1^st^ visit | Underweight (18.5 ≧x)  (N = 2626) | Normal (25 ≧ x > 18.5)  (N = 162,523) | Overweight (30 ≧ x > 25)  (N = 212097) | Obesity (30 > x)  (N = 122153) |
| --- | --- | --- | --- | --- |
| Number of patients with diabetes (With:Without)* | 35:2575 | 3076:158960 | 8976:202284 | 13991:107362 |
| Length of diabetes* | 14.50 (SD:10.98, N = 28) | 12.48 (SD:13.42, M = 2713) | 9.25 (SD:11.14, N = 8399) | 7.79 (SD:930, N = 13158) |
| Frequency of reports of complications (none:1: 2)** | 34:1:0 | 2881:193:2 | 8421:548:7 | 13123:851:16 |
| 3rd visit (who attended both third and fourth assessment) | Underweight (18.5≧x)  N = 19 | Normal (25≧x > 18.5)  N = 1222 | Overweight (30≧x > 25)  N = 1201 | Obesity (30 > x)  N = 484 |
| Number of patients with diabetes (With:Without)* | 0:19 | 26:1190 | 57:1140 | 44:435 |
| Length of diabetes* | - | 12.00 (SD:11.10, N = 24) | 10.02 (SD:10.69, N57) | 5.95 (SD:5.59, N = 41) |
| Frequency of number of reports of complications (none:1: 2)** | - | 25:1:0 | 51:6:0 | 39:5:0 |

* Note there are subjects with no information available.

** Sum of the reports of the existence of self-reported diabetes-related eye disease in either eye (UK Biobank data field ID:5890, self-reported diabetic neuropathy/ulcers (UK Biobank data field ID:20002), diabetes insipidus (UK Biobank data field ID:20002), self-reported nephropathy (UK Biobank data field ID:20002).

**Supplemental Table 10**. The effects of diagnosis of diabetes on outcome measures in the main analyses, and how the statistical values of effects of BMI on outcome measures change when the covariate of diagnosis of diabetes is excluded.

|  | Effects of diagnosis of diabetes at baseline in the main analysis | | | Effects of BMI in the  main analysis | | | Effects of BMI in the analysis without including the covariate of diabetes at baseline | | | |
| --- | --- | --- | --- | --- | --- | --- | --- | --- | --- | --- |
|  | Multiple regression analysis | | | | | | | | |  |
|  | p | t | beta | p | t | beta | p | t | beta | N |
| Fluid intelligence change | 0.924 | -0.096 | -7.6*10^-4^ | 0.283 | −1.074 | -0.010 | 0.309 | -1.017 | -0.008 | 13422 |
| Reaction time change | 2.1*10^-7^ | 5.190 | 0.024 | 0.048 | 1.978 | 0.009 | 0.011 | 2.544 | 0.012 | 39826 |
| Visuospatial memory (error) change | 0.969 | 0.038 | 1.5*10^-4^ | 0.000001 | -4.854 | -0.019 | 0.000001 | -4.881 | -0.019 | 39229 |
| Depressive symptoms change | 0.001 | 3.353 | 0.015 | 2.6*10^-15^ | 7.911 | 0.036 | 6.2*10^-17^ | 8.365 | 0.038 | 38248 |
| Total gray matter volume | 0.312 | -1.012 | -0.006 | 0.497 | 0.680 | 0.004 | 0.561 | 0.582 | 0.004 | 1253 |
| Total white matter volume | 0.472 | -0.720 | -0.005 | 0.045 | 2.005 | 0.013 | 0.053 | 1.940 | 0.013 | 1253 |
| Total FA | 0.941 | 0.074 | 0.001 | 0.013 | 2.480 | 0.021 | 0.012 | 2.504 | 0.021 | 1243 |
| Total MD | 0.601 | -0.524 | -0.006 | 3.3*10^-8^ | -5.561 | -0.062 | 2.2*10^-8^ | -5.650 | -0.062 | 1243 |
| Total AD | 0.506 | -0.665 | -0.009 | 3.8*10^-8^ | -5.537 | -0.075 | 2.1*10^-8^ | -5.638 | -0.076 | 1243 |
| Total RD | 0.727 | -0.349 | -0.003 | 5.1*10^-8^ | -5.481 | -0.054 | 3.5*10^-8^ | -5.552 | -0.054 | 1243 |
| Total ICVF | 0.453 | -0.750 | -0.010 | 1.4*10^-8^ | 5.716 | 0.079 | 1.4*10^-8^ | 5.670 | 0.078 | 1243 |
| Total ISOVF | 0.475 | -0.715 | -0.011 | 0.440 | -0.772 | -0.012 | 0.394 | -0.852 | -0.013 | 1243 |
| Total OD | 0.814 | -0.236 | -0.004 | 9.9*10^-11^ | 6.526 | 0.124 | 9.0*10^-11^ | 6.540 | 0.124 | 1243 |
|  | Cox hazard regression model | | | | | | | | |  |
|  | p | Wald | Exp(B) | p | Wald | Exp(B) | p | Wald | Exp(B) | N |
| Dementia (analyses with BMI as a categorical variable) | 4.1*10^-17^ | 70.713 | 1.914 | 9.9*10^-5^ | 21.120 | - | 0.001 | 16.230 | - | 400580 |

Note, in analyses of psychological measures, dependent variables were changes of scores of outcome measurements, but in imaging analyses of imaging measures, dependent variables were values at the post measurement. In both of the cases, P values and t values are same as long as outcome values at the pre-measurement are adjusted in analyses. Also, the information of sample size of main analyses ysis was provided in the main analyses.

**Supplemental Table 11.** Differences in outcomes between subjects with and without overweight/obesity and diabetes analyzed by ANCOVAs.

|  | Adjusted value of non-overweight/obese, non-diabetes  (Adjusted value,  95%CI  N) | Adjusted values of non-overweight/obese, diabetes  (Adjusted value,  95%CI  N) | Adjusted value of overweight/obese, non-diabetes  (Adjusted value,  95%CI  N) | Adjusted values of overweight/obese, diabetes  (Adjusted value,  95%CI  N) | P  (Overall group difference,  each significant group difference)* |
| --- | --- | --- | --- | --- | --- |
| Fluid intelligence change | −0.011  −0.055–0.033  5020 | −0.237  −0.667–0.194  49 | −0.066  −0.101–−0.032  7960 | −0.036  −0.200–0.128  357 | 0.217 |
| Reaction time change | 57.83  56.33–59.33  15382 | 66.55  51.36–81.73  143 | 59.58  58.38–60.78  23356 | 76.78  70.56–82.99  901 | 1.87 × 10^−7^  nOnD < OD(8.46 × 10^−9^)  OnD < OD(9.23 × 10^−8^) |
| Visuospatial memory (error) change | −0.0274  −0.0738–0.0189  15122 | 0.0095  −0.4618–0.4807  139 | −0.1608  −0.1978–−0.1238  23041 | −0.1985  −0.3900–−0.0069  889 | 0.000002  nOnD > OnD(0.000015) |
| Depressive symptoms change | −0.2053  −0.2297–−0.1810  14709 | 0.0009  −0.2375–0.2393  146 | −0.1181  −0.1375–−0.987  22486 | 0.0725  −0.0276–0.1726  874 | 2.69 × 10^−8^  nOnD < OnD(6.92 × 10^−8^)  nOnD < OD(1.59 × 10^−7^)  OnD < OD(0.00024) |
| Total gray matter volume | 616910  615857–617963  515 | 618844  611327–626361  10 | 618054  617148–618960  679 | 615545  612119–618972  49 | 0.253 |
| Total white matter volume | 543090  541883–544296  515 | 544141  535529–552753  10 | 543939  542900–544977  679 | 542388  538470–546305  49 | 0.690 |
| Total FA | 0.4962  0.4958–0.4966  513 | 0.4964  0.4935–0.4994  10 | 0.4965  0.4962–0.4969  671 | 0.4967  0.4954–0.4980  49 | 0.691 |
| Total MD | 74.44  74.36–74.52  513 | 74.08  73.51–74.64  10 | 74.16  74.09–74.23  671 | 74.13  73.88–74.39  49 | 0.000003  nOnD > OnD(1.85 × 10^−7^)  nOnD > OD(0.025) |
| Total AD | 103.35  103.26–103.45  513 | 102.90  102.21–103.58  10 | 102.99  102.91–103.07  671 | 102.94  102.63–103.25  49 | 7.06 × 10^−7^  nOnD > OnD(4.77 × 10^−8^)  nOnD > OD(0.014) |
| Total RD | 59.99  59.91–60.06  513 | 59.68  59.16–60.19  10 | 59.74  59.68–59.80  671 | 59.73  59.50–59.97  49 | 0.000011  nOnD > OnD(6.67 × 10^−7^)  nOnD > OD(0.045) |
| Total ICVF | 52486  52396–52577  513 | 52610  51964–53256  10 | 52653  52575–52731  671 | 52565  52270–52859  49 | 0.064  nOnD < OnD(0.007) |
| Total ISOVF | 4967  4921–5014  513 | 4857  4524–5189  10 | 4883  4842–4923  671 | 4854  4702–5005  49 | 0.054  nOnD > OnD(0.008) |
| Total OD | 31345  31300–31389  513 | 31506  31191–31820  10 | 31482  31444–31521  671 | 31457  31314–31600  49 | 0.000120  nOnD < OnD(6.0 × 10^−6^) |
| Cox hazard regression model | non-obesity, non-diabetes  (raw number of incident dementia/group, HR) | non-obesity, diabetes  (raw number of incident dementia/group, HR, 95%CI) | obesity, non-diabetes  (raw number of incident dementia/group, HR, 95%CI) | obesity,  diabetes  (raw number of incident dementia/group, HR, 95%CI) | p  (Overall group difference,  each significant group difference) × |
| Dementia | 464/131722  reference | 30/2286  1.955  1.345–2.843 | 898/248535  0.800  0.713–0.898 | 201/17239  1.517  1.269–1.814 | 1.63 × 10^−17^  nOnD < nOD(0.000449)  nOnD > OnD(0.000144)  nOnD < OD(0.000005)  nOD > OnD(0.000002)  OnD < OD(4.66 × 10^−15^) |

Note, in analyses of psychological measures, dependent variables were changes of scores of outcome measurements, but in imaging analyses of imaging measures, dependent variables were values at the post measurement. P values are same as long as outcome values at thepre measurement are adjusted in analyses. Also, the information of sample size of main analyses ysis was provided in the main analyses.

*nOnD = non-overweight/obese, non-diabetes, nOD = non-overweight/obese, diabetes, OnD = overweight/obese, non-diabetes, OD = overweight/obese, diabetes.

**Supplemental Table 12**. The effects of baseline hyperlipidemia on outcome measures in supplemental analyses including hyperlipidemia at baseline as covariate and changes from the main analyses in BMI effects on outcome measures.

|  | Effects of BMI in the  main analysis | | | Effects of BMI in the analysis including hyperlipidemia at baseline as covariate | | | Effects of hyperlipidemia at baseline in the analysis including hyperlipidemia at baseline as covariate | | | |
| --- | --- | --- | --- | --- | --- | --- | --- | --- | --- | --- |
| Multiple regression analysis | | | | | | | | | |  |
|  | p | t | beta | p | t | beta | p | t | beta | N |
| Fluid intelligence change | 0.283 | −1.074 | −0.010 | 0.284 | −1.071 | −0.009 | 0.961 | −0.049 | −4.8 × 10^−4^ | 13409 |
| Reaction time change | 0.048 | 1.978 | 0.009 | 0.051 | 1.953 | 0.009 | 0.579 | 0.555 | 0.003 | 39782 |
| Visuospatial memory (error) change | 0.000001 | −4.854 | −0.019 | 0.000001 | −4.870 | −0.019 | 0.653 | 0.449 | 0.002 | 39191 |
| Depressive symptoms change | 2.6 × 10^−15^ | 7.911 | 0.036 | 1.1 × 10^−14^ | 7.728 | 0.035 | 0.000009 | 4.445 | 0.024 | 38215 |
| Total gray matter volume | 0.497 | 0.680 | 0.004 | 0.470 | 0.723 | 0.005 | 0.469 | −0.725 | −0.005 | 1253 |
| Total white matter volume | 0.045 | 2.005 | 0.013 | 0.040 | 2.052 | 0.014 | 0.403 | −0.837 | −0.006 | 1253 |
| Total FA | 0.013 | 2.480 | 0.021 | 0.011 | 2.541 | 0.021 | 0.279 | −1.083 | −0.009 | 1243 |
| Total MD | 3.3 × 10^−8^ | −5.561 | −0.062 | 2.9 × 10^−8^ | −5.582 | −0.062 | 0.577 | 0.557 | 0.007 | 1243 |
| Total AD | 3.8 × 10^−8^ | −5.537 | −0.075 | 3.7 × 10^−8^ | −5.540 | −0.075 | 0.796 | 0.258 | 0.004 | 1243 |
| Total RD | 5.1 × 10^−8^ | −5.481 | −0.054 | 4.4 × 10^−8^ | −5.509 | −0.054 | 0.506 | 0.666 | 0.007 | 1243 |
| Total ICVF | 1.4 × 10^−8^ | 5.716 | 0.079 | 1.3 × 10^−8^ | 5.721 | 0.079 | 0.777 | −0.284 | −0.004 | 1243 |
| Total ISOVF | 0.440 | −0.772 | −0.012 | 0.430 | −0.790 | −0.012 | 0.735 | 0.338 | 0.006 | 1243 |
| Total OD | 9.9 × 10^−11^ | 6.526 | 0.124 | 9.3 × 10^−11^ | 6.536 | 0.125 | 0.684 | −0.407 | −0.008 | 1243 |
| Cox hazard regression model | | | | | | | | | |  |
|  | p | Wald | Exp(B) | p | Wald | Exp(B) | p | Wald | Exp(B) | N |
| Dementia (analyses with BMI as a categorical variable) | 9.9 × 10^−5^ | 21.120 | - | 4.4 × 10^−5^ | 22.828 | - | 0.001 | 11.727 | 1.256 | 399782 |

Note, in analyses of psychological measures, dependent variables were changes of scores of outcome measurements, but in imaging analyses of imaging measures, dependent variables were values at the post measurement. P values are same as long as outcome values at the pre-measurement are adjusted in analyses. Also, the information of sample size of main analyses ysis was provided in the main analyses.

**Supplemental Table 13**. Impact of excluding subjects with comorbidities on the effects of BMI on outcome measures

|  | Effects of BMI in the  main analysis | | | Effects of BMI in the analysis excluding subjects with 11 types of comorbidities | | | | Effects of BMI in the analysis excluding subjects with cancer and any “other serious medical conditions” | | | | |
| --- | --- | --- | --- | --- | --- | --- | --- | --- | --- | --- | --- | --- |
|  | p | t | beta | p | t | beta | N | p | t | beta | N |  |
| Fluid intelligence change | 0.283 | −1.074 | −0.010 | 0.146 | −1.455 | 0.015 | 7746 | 0.255 | −1.139 | −0.010 | 10547 |  |
| Reaction time change | 0.048 | 1.978 | 0.009 | 0.059 | 1.885 | 0.011 | 23456 | 0.014 | 2.462 | 0.013 | 31721 |  |
| Visuospatial memory (error) change | 0.000001 | −4.854 | −0.019 | 0.05 | −1.961 | −0.010 | 23161 | 0.001 | −3.230 | −0.014 | 31272 |  |
| Depressive symptoms change | 2.6 × 10^−15^ | 7.911 | 0.036 | 1.40 × 10−^7^ | 5.267 | 0.031 | 22485 | 2.8 × 10^−11^ | 6.659 | 0.034 | 30492 |  |
| Total gray matter volume | 0.497 | 0.680 | 0.004 | 0.016 | 2.426 | 0.020 | 666 | 0.159 | 1.409 | 0.010 | 1007 |  |
| Total white matter volume | 0.045 | 2.005 | 0.013 | 0.902 | 0.123 | 0.001 |  | 0.319 | 0.996 | 0.007 | 1007 |  |
| Total FA | 0.013 | 2.480 | 0.021 | 0.021 | 2.305 | 0.025 | 666 | 0.032 | 2.142 | 0.020 | 999 |  |
| Total MD | 3.3 × 10^−8^ | −5.561 | −0.062 | 2.9 × 10^−7^ | −5.187 | −0.077 | 661 | 7.0 × 10^−6^ | −4.530 | −0.055 | 999 |  |
| Total AD | 3.8 × 10^−8^ | −5.537 | −0.075 | 6.3 × 10^−7^ | –5.034 | –0.093 | 661 | 5.0 × 10^−6^ | −4.609 | −0.069 | 999 |  |
| Total RD | 5.1 × 10^−8^ | −5.481 | −0.054 | 3.7 × 10^−7^ | −5.138 | −0.067 | 661 | 1.2 × 10^−5^ | −4.406 | −0.047 | 999 |  |
| Total ICVF | 1.4 × 10^−8^ | 5.716 | 0.079 | 0.009 | 2.626 | 0.030 | 661 | 0.006 | 2.731 | 0.026 | 999 |  |
| Total ISOVF | 0.440 | −0.772 | −0.012 | 4.8 × 10^−4^ | −3.508 | −0.075 | 661 | 0.015 | −2.445 | −0.041 | 999 |  |
| Total OD | 9.9 × 10^−11^ | 6.526 | 0.124 | 4.5 × 10^−4^ | 3.525 | 0.074 | 661 | 1.1 × 10^−4^ | 3.890 | 0.069 | 999 |  |
|  | p | Wald | Exp(B) | p | Wald | Exp(B) | N | P | Wald | Exp(B) | N |  |
| Dementia (analyses with BMI as a categorical variable) | 9.9 × 10^−5^ | 21.120 | - | 0.012 | 10.906 | - | 203368 | 0.031 | 88.70 | - | 298708 |  |

Note, in analyses of psychological measures, dependent variables were changes of scores of outcome measurements, but in imaging analyses of imaging measures, dependent variables were values at the post measurement. P values are same as long as outcome values at the pre-measurement are adjusted in analyses.
